# Transcriptomic Profiling of Human Pluripotent Stem Cell-derived Retinal Pigment Epithelium over Time

**DOI:** 10.1016/j.gpb.2020.08.002

**Published:** 2020-12-08

**Authors:** Grace E. Lidgerwood, Anne Senabouth, Casey J.A. Smith-Anttila, Vikkitharan Gnanasambandapillai, Dominik C. Kaczorowski, Daniela Amann-Zalcenstein, Erica L. Fletcher, Shalin H. Naik, Alex W. Hewitt, Joseph E. Powell, Alice Pébay

**Affiliations:** 1 Department of Anatomy and Neuroscience, The University of Melbourne, Parkville, VIC 3010, Australia; 2 Department of Surgery, The University of Melbourne, Parkville, VIC 3010, Australia; 3 Centre for Eye Research Australia, Royal Victorian Eye and Ear Hospital, East Melbourne, VIC 3002, Australia; 4 Garvan Weizmann Centre for Cellular Genomics, Garvan Institute of Medical Research, The Kinghorn Cancer Centre, Darlinghurst, NSW 2010, Australia; 5 Single Cell Open Research Endeavour, The Walter and Eliza Hall Institute of Medical Research, Parkville, VIC 3052, Australia; 6 Immunology Division, The Walter and Eliza Hall Institute of Medical Research, Parkville, VIC 3052, Australia; 7 Department of Medical Biology, The University of Melbourne, Parkville, VIC 3010, Australia; 8 School of Medicine, Menzies Institute for Medical Research, University of Tasmania, Hobart, TAS 7005, Australia; 9 UNSW Cellular Genomics Futures Institute, School of Medical Sciences, University of New South Wales, Sydney, NSW 2052, Australia

**Keywords:** Human embryonic stem cell, Human pluripotent stem cell, Retinal pigment epithelium, Single-cell RNA sequencing, Ageing

## Abstract

**Human pluripotent stem cell** (hPSC)-derived progenies are immature versions of cells, presenting a potential limitation to the accurate modelling of diseases associated with maturity or age. Hence, it is important to characterise how closely cells used in culture resemble their native counterparts. In order to select appropriate time points of **retinal pigment epithelium** (RPE) cultures that reflect native counterparts, we characterised the transcriptomic profiles of the hPSC-derived RPE cells from 1- and 12-month cultures. We differentiated the **human embryonic stem cell** line H9 into RPE cells, performed single-cell RNA-sequencing of a total of 16,576 cells to assess the molecular changes of the RPE cells across these two culture time points. Our results indicate the stability of the RPE transcriptomic signature, with no evidence of an epithelial–mesenchymal transition, and with the maturing populations of the RPE observed with time in culture. Assessment of Gene Ontology pathways revealed that as the cultures age, RPE cells upregulate expression of genes involved in metal binding and antioxidant functions. This might reflect an increased ability to handle oxidative stress as cells mature. Comparison with native human RPE data confirms a maturing transcriptional profile of RPE cells in culture. These results suggest that long-term *in vitro* culture of RPE cells allows the modelling of specific phenotypes observed in native mature tissues. Our work highlights the transcriptional landscape of hPSC-derived RPE cells as they age in culture, which provides a reference for native and patient samples to be benchmarked against.

## Introduction

The retinal pigment epithelium (RPE) is a monolayer of post-mitotic, pigmented polarised cells that is key to the health and function of photoreceptors and underlying vasculature. In particular, the RPE protects the retina against photo-oxidation and phagocytoses photoreceptor outer segments. The RPE is also essential to the immune privilege of the eye, as it physically contributes to the blood–retina barrier and also expresses molecules repressing the migration of immune cells into the retina [1]. In the human retina, ageing is associated with vision decline and delayed dark adaptation, both of which are direct consequences of tissue stress and retinal damage [2]. It is hypothesised that over time, oxidative stress leads to the death of retinal neurons, a decrease in the number of RPE cells, an accumulation of the toxic waste lipofuscin within the RPE, and an accumulation of basal toxic deposits called drusen underneath the RPE [2]. Together, these events contribute to a loss of homeostasis and low-grade inflammation within the retina [2]. Although it is clear that the RPE is key to the health of the retina, the precise molecular mechanisms underlying its ageing are not well understood.

Human pluripotent stem cells (hPSCs) have the ability to propagate indefinitely *in vitro* and give rise to any cell types in the body, including cells that form the retina. Various protocols have been described to differentiate hPSCs into RPE cells [3], [4], [5], [6], [7], [8]. RPE cells are generally assayed after a few weeks of differentiation, at which stage they demonstrate similarity to their human native counterparts, in terms of morphology/expression of key proteins, functions, and expression profiles, however with a profile closer to a foetal stage than adult stage [4], [9], [10]. Interestingly, the transcriptome profile of hPSC-derived RPE cells as they age in culture is unknown. To date, most RNA-sequencing (RNA-seq) studies of RPE cells have been performed on bulk samples. Yet, the ability to sequence individual cells provides a powerful tool to precisely uncover potential heterogeneity in cell population, especially as these cells develop and mature *in vitro*. Here, we used single-cell RNA-seq (scRNA-seq) of hPSC-derived RPE cells maintained in culture for 1 month or 12 months to assess the impact of time on the RPE transcriptome and whether genetic hallmarks of maturation can be observed over time. A short-time differentiation (1 month) was chosen as it represents a time point routinely used in *in vitro* assays of RPE [3]. A prolonged time course of differentiation (12 months) was chosen as its characterization could be subsequently used for comparison with other retinal cell differentiation methods, in particular of retinal organoids and photoreceptors, for which differentiation and relative maturity are obtained after prolonged time in culture and would thus be present at that later time point [11], [12], [13], [14].

## Results

### scRNA-seq profiles the transcriptomes of 16,576 cells

**Figure 1:**
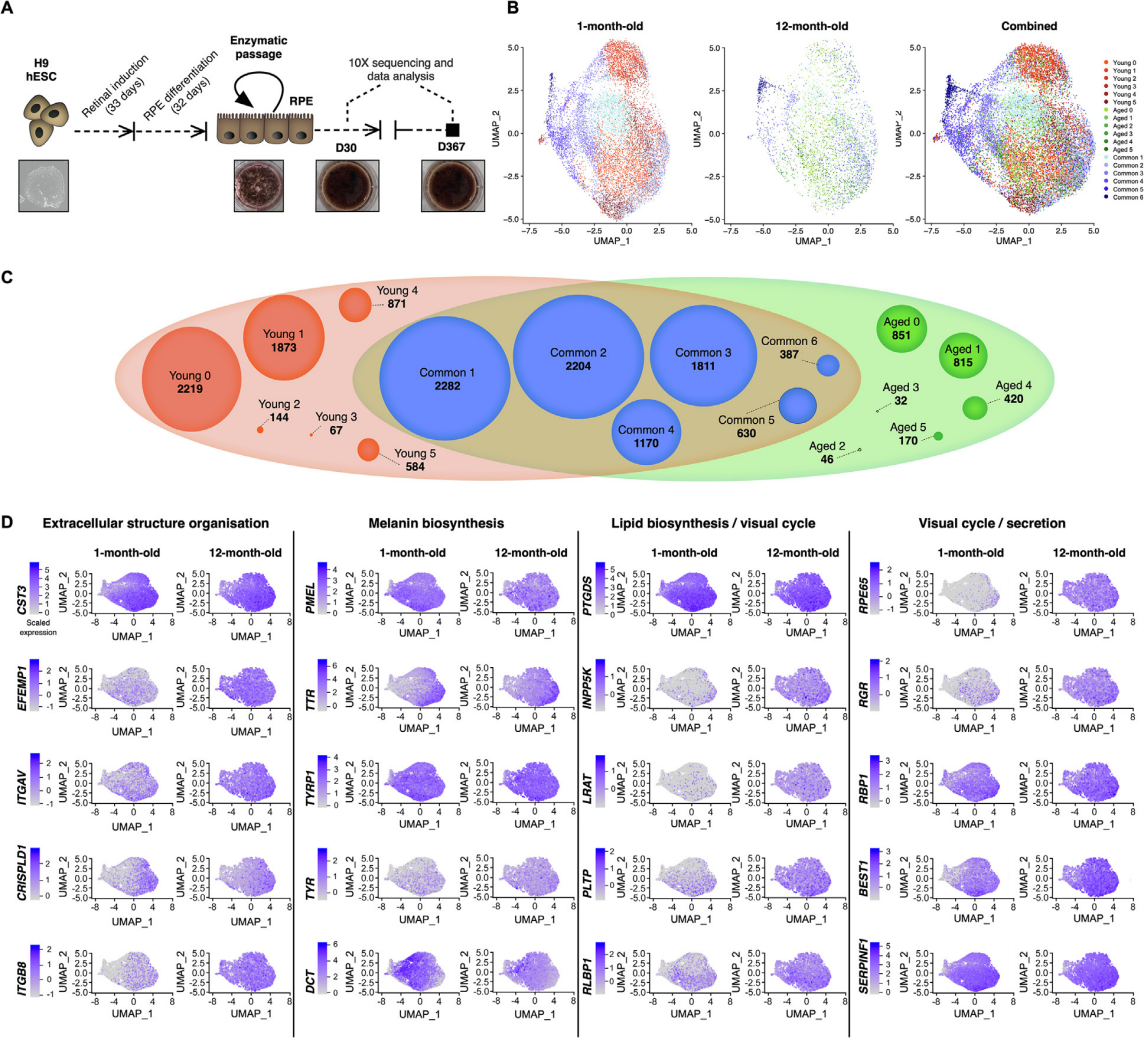
**scRNA-seq transcriptome profiling of hPSC-derived RPE cells reveals 18 subpopulations** **A.** Schematic representations of the experimental flow. **B.** UMAP of single-cell expression profile from 16,576 cells, clustered into 18 subpopulations, split by condition (1-month-old and 12-month-old) and combined. **C.** Cluster grouping represented by a Venn diagram, identifying 18 subpopulations, showing Young (red), Aged (green), and their common subpopulations (blue). Number of cells for each subpopulation is indicated in bold below the subpopulation name. **D.** UMAP of canonical RPE markers in 1-month-old and 12-month-old cultures, organised by cellular functions: extracellular structure organisation (*CST3*, *EFEMP1*, *ITGAV, CRISPLD1*, and *ITGB8*); melanin biosynthesis (*PMEL*, *TTR*, *TYRP1*, *TYR*, and *DCT*), lipid biosynthesis (*PTGDS* and *INPP5K),* visual cycle (*LRAT, PLTP, RLBP1*, *RPE65*, *RGR*, *RBP1*, and *BEST1*), as well as secretion (*SERPINF1*). Levels of gene expression per cell (percentage expressed) are shown with colour gradients. scRNA-seq, single-cell RNA sequencing; hPSC, human pluripotent stem cell; RPE, retinal pigment epithelium; UMAP, Uniform Manifold Approximation and Projection for Dimension Reduction.

The human embryonic stem cell (hESC) line H9 was differentiated to RPE cells following the protocol described in the Materials and methods section. To generate a transcriptional map of the RPE cells reflecting time in culture, RPE cells from the same culture and original passaging were isolated after 1 month or 12 months of differentiation, dissociated to single cells, and processed to generate libraries for scRNA-seq analysis (Figure 1A). The capture of single-cell library from the 1-month-old culture detected 12,873 cells at the mean read depth of 40,499 reads per cell, while the capture from the 12-month-old culture detected 4850 cells at the mean read depth of 114,503 reads per cell (Table S1). Both datasets were subjected to cell-specific quality control, where 510 cells and 637 cells were removed from the 1-month-old and 12-month-old culture datasets, respectively. The remaining 16,576 cells were retained for further analysis.

We compared variations in the transcriptomic profiles between the 1-month-old and 12-month-old samples (Figure 1B and C) to identify potential changes in phenotypes upon ageing of RPE cells *in vitro*, by analysing differential expression. A range of RPE markers were observed as conserved between both time points (Figure 1D). In particular, canonical RPE markers [15] associated with extracellular structure organisation (*CST3*, *EFEMP1*, *ITGAV*, *CRISPLD1*, and *ITGB8*), melanin biosynthesis (*PMEL*, *TTR*, *TYRP1*, *TYR*, and *DCT*), lipid biosynthesis (*PTGDS* and *INPP5K),* visual cycle (*LRAT*, *PLTP*, *ABHD2/RLBP1*, *RPE65*, *RGR*, *RBP1*, and *BEST1*), and secretion (*SERPINF1*) were expressed at both time points (Figure 1D).

### Clustering analysis highlights 12 subpopulations of RPE cells

**Figure 2:**
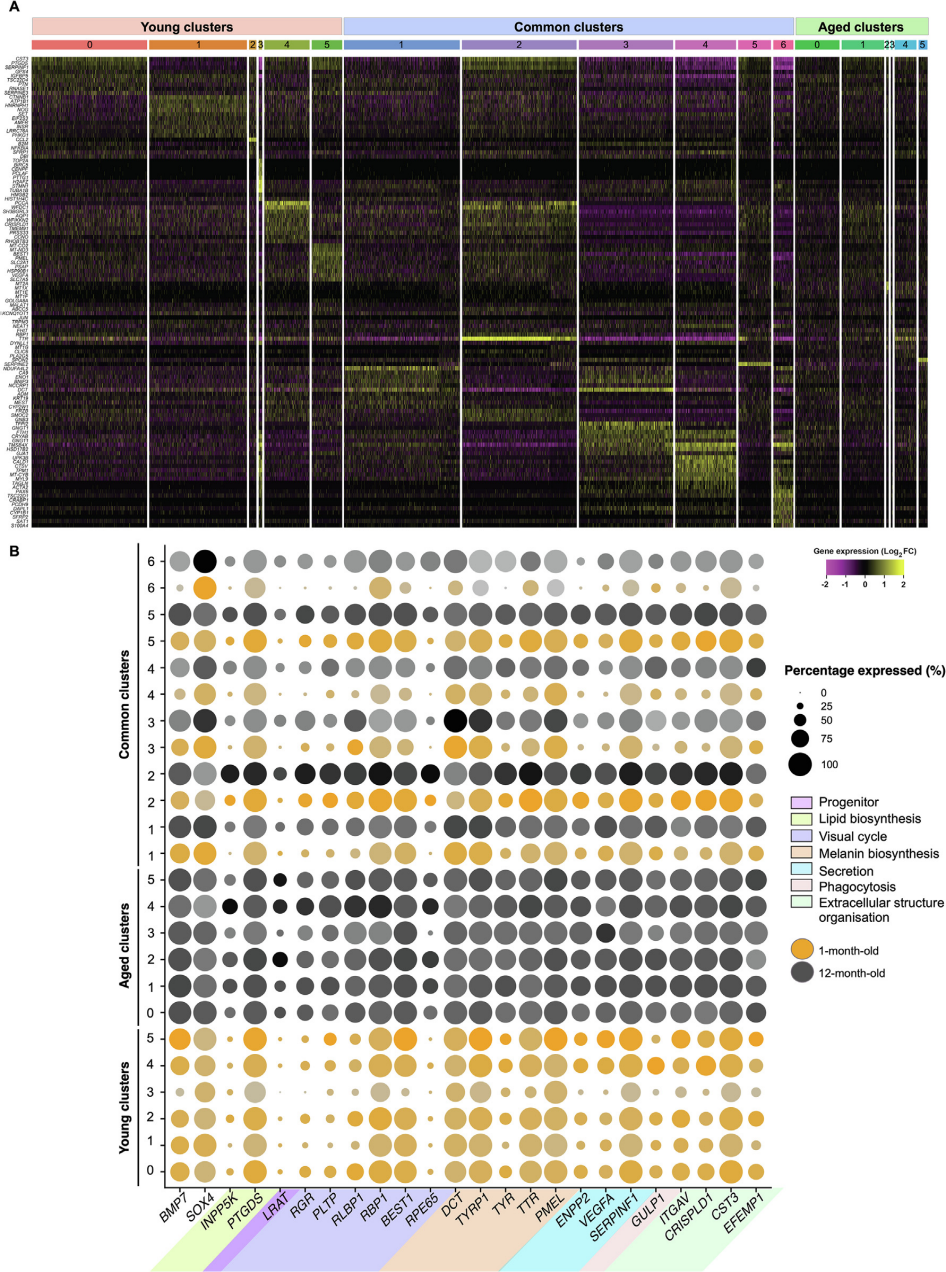
**Characterisation of hPSC-derived RPE populations** **A.** Heatmap showing the most conserved markers (gene symbols are indicated on the left side) in all individual cells in each of the 18 subpopulations (indicated on top, with colours matching those of subpopulations shown in Figure 1C). Gene expression levels were scaled and presented as average of Log_2_-transformed FC. **B.** Dotplot representation of single-cell expression profile from 1-month-old and 12-month-old cells for selected gene markers, representative of progenitor cells, or RPE with genes linked to RPE functions. Populations arising from 1-month-old cultures are represented in orange and those from 12-month-old cultures in black. Levels of gene expression per cell are shown with colour gradients, and frequencies of cells expressing the respective gene (percentage expressed) are shown with size of dots. RPE markers are coloured according to their cellular functions. FC, fold change.

Clustering analysis was performed independently for samples obtained at the two time points and identified 12 subpopulations (clusters) in each sample (Figure 1C; Table S2). After integrating with anchors identified with a method described previously [16], MetaNeighbor was used to match common subpopulations across both samples [17], denoted as “Common”. Clusters unique to 1-month-old and 12-month-old samples are denoted as “Young” and “Aged”, respectively (Figure 1C). In total, 18 subpopulations were identified, including six common subpopulations (Common 1–6; 8484 cells; Table S2), six subpopulations exclusive to the 1-month-old dataset (Young 0–5; 5758 cells; Table S2), and six subpopulations exclusive to the 12-month-old dataset (Aged 0–5; 2334 cells; Table S2). Cell counts per cluster (Figure 1C; Table S2) and the top conserved markers for each distinct cluster (Figure 2A; Tables S3–S5) were identified. Clusters were visualised using the Uniform Manifold Approximation and Projection (UMAP) plots (Figure 1B). In total, 3070 cells were considered singletons. These cells had fewer connections with similar cells (neighbours) relative to the rest of the cell population and could not be assigned to a subpopulation. Cluster 0 in the 1-month-old dataset (Young 0) comprises 2219 cells, accounting for 18% of all 1-month-old cells, whereas Cluster 0 in the 12-month-old dataset (Aged 0) comprises 851 cells, accounting for 20% of all 12-month-old cells (Table S2). There were more singleton cells in the Young population as we captured more cells from this group. We assessed the expression profile of genes characteristic of progenitors (*BMP7* and *SOX4*) and canonical RPE genes [15] across all subpopulations, both in terms of frequency and intensity of expression (Figure 2B; Tables S3–S5). These genes are linked to lipid biosynthesis (*INPP5K* and *PTGDS*), visual cycle (*LRAT, RGR*, *PLTP*, *RLBP1/CRALBP*, *RBP1/CRBP1*, *BEST1*, and *RPE65*), melanin biosynthesis (*DCT*, *TYRP1*, *TYR*, *TTR*, and *PMEL*); secretion (*ENPP2*, *VEGFA*, and *SERPINF1*), phagocytic activity (*GULP1*), and extracellular structure organisation (*ITGAV*, *CRISPLD1*, *CST3*, and *EFEMP1*). Most canonical RPE genes were expressed across most populations, although many were found at lower levels in the 1-month-old cells, confirming the purity of the RPE cell cultures over time (Figure 2B). As these transcripts are associated with stages of RPE maturity, our data indicate that all subpopulations are of RPE lineage, potentially at various stages of differentiation and maturation.

### Most cells share common transcriptomic profiles suggestive of maturing RPE cells

**Figure 3:**
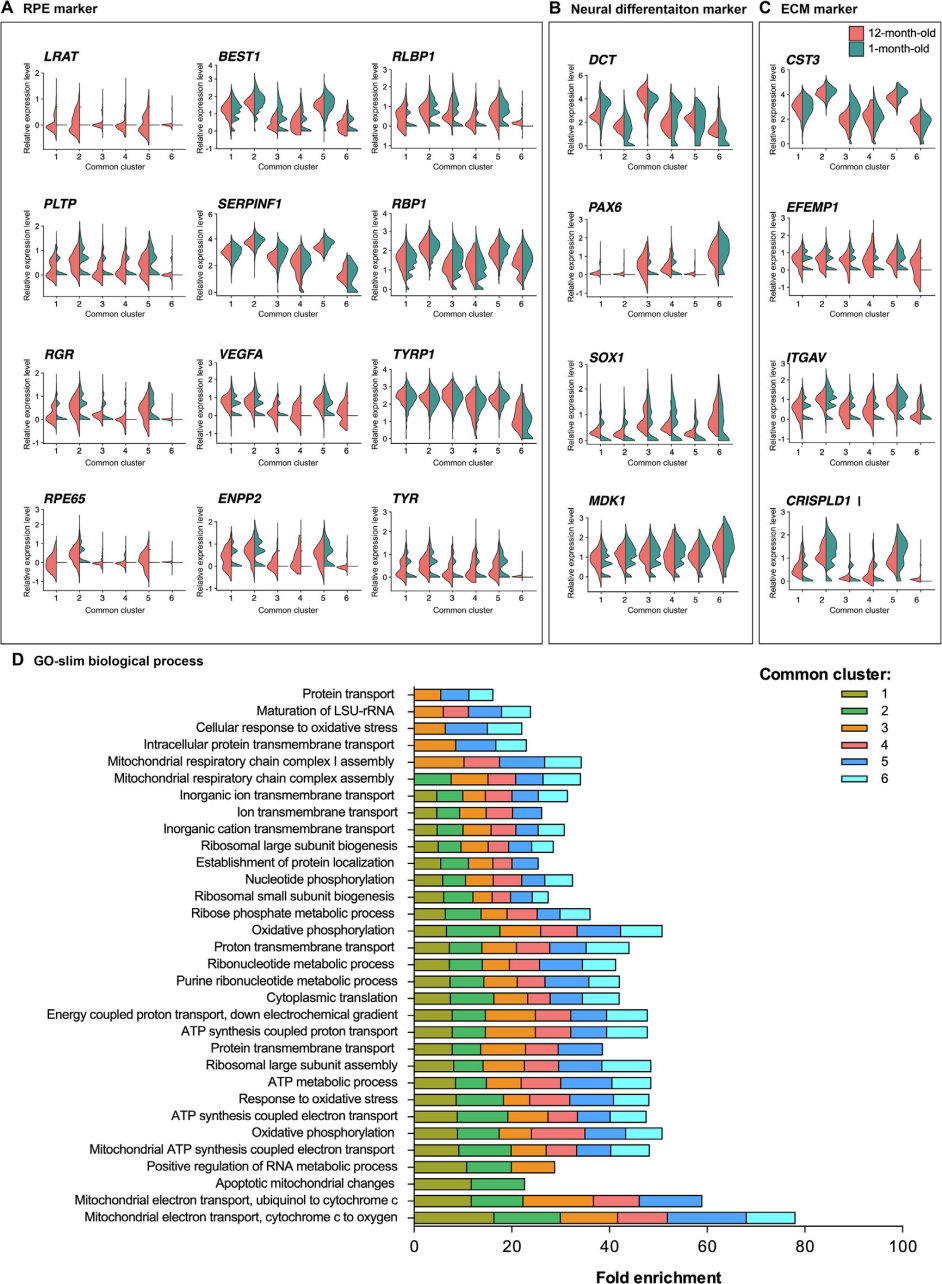
**Expression patterns of selected conserved markers and GO pathway in the hPSC-derived RPE cells** Expression values are measured as normalised UMI counts. **A.** Violin plot of selected conserved markers in each Common subpopulation characteristic of the RPE. **B.** Violin plot of selected conserved markers in each Common subpopulation characteristic of the neural differentiation. **C.** Violin plot of selected conserved markers in each Common subpopulation characteristic of the ECM. The plots describe the distribution and relative expression of each transcript in each common subpopulation, with separation of cells belonging to the 1-month-old (blue) and 12-month-old (brown) cultures. **D.** PANTHER GO-slim (biological process) pathways associated with each of the Common subpopulations (Common 1–6; colour-coded) identified via over-representation analysis. Association is measured by fold enrichment, that is calculated from the number of genes observed, divided by the expected number of genes to be present by chance. UMI, unique molecular identifier; ECM, extracellular matrix.

We then performed differential gene expression analysis and pathway enrichment analysis to characterise the molecular signature of these subpopulations. Of the cells examined, more than half (8484 cells) were clustered into six Common subpopulations that intersect the 1-month-old and 12-month-old cell cultures (Figure 1B and C), indicating a large shared transcriptional profile between the two conditions. Some commonalities and differences were observed between the Common samples arising from the 1-month-old and 12-month-old cultures. A range of RPE markers was observed as conserved between samples collected at both time points (Figure 2B). In particular, RPE markers associated with melanin biosynthesis (*MITF*, *PMEL*, *TTR*, *TYR*, *TYRP1*, and *DCT*), extracellular structure organisation (*EFEMP1*, *CST3*, *CRISPLD1*, *ITGAV*, and *ITGB8*), secretion (*SERPINF1* and *VEGFA*), visual cycle (*RPE65*, *BEST1*, *RBP1*, *RLBP1*, *PLTP*, *RGR*, and *LRAT*), tight junctions (*TJP1*), phagocytic activity (*GULP1*), and lipid biosynthesis (*PTGDS*, *CYP27A1*, *INPP5K*, *PLA2G16*, and *PLCE1*) were conserved in all or some of the subpopulations (Table S3). Expression of some genes related to RPE maturity did not appear to differ between the 1-month-old and 12-month-old RPE cells. These include *RBP1*, *TYRP1*, and *SERPINF1*, demonstrating that some genes encoding proteins necessary for retinoid-cycle binding, melanin biosynthesis and secretory are expressed in early RPE development (Figure 2B). More heterogeneity was observed in the expression of RPE markers *RGR*, *PLTP*, *RLBP1*, *BEST1*, *ENPP2*, *VEGFA*, and *TYR* in the 1-month-old RPE cells relative to the 12-month-old RPE cells, in which expression of these genes was generally high and stable (Figures 1D and 2B). Expression of *LRAT* and *RPE65* was predominantly observed in the 12-month-old RPE cells, with the exception of low expression of *RPE65* in the 1-month-old sample for the Common 2 subpopulation. This suggests that the catabolic machinery converting all*-trans*-retinol into all-*trans*-retinyl ester (LRAT) and 11-*cis*-retinol (RPE65) for phototransduction is expressed at low levels in the 1-month-old RPE samples but becomes more comprehensive as cells age in culture (Figure 2C and D). Variations in the pattern of gene expression were observed between cells identified from the 1-month-old or 12-month-old cultures within each subpopulation (Figure 3A–C). Genes involved in neural differentiation, including *DCT*, *PAX6*, *SOX1*, and *MDK*, exhibited notable differences in expression between the 1-month-old and 12-month-old samples (Figure 3B). Similar patterns were also observed in genes involved in the extracellular matrix (ECM) formation and maintenance, including *CST3*, *EFEMP1*, *ITGAV*, and *CRISPLD1* (Figure 3C), which were more heterogeneous in the 1-month-old samples, suggesting transitional changes in ECM markers during early RPE differentiation. Examples of genes characteristics of RPE, neural differentiation, and ECM are illustrated in Figure 3A–C, respectively.

The common subpopulation 1 (Common 1, 2282 cells) was characterised by 891 conserved markers identified (*P* < 0.74) (Table S3). The most highly conserved markers include *NDUFA4L2* (a gene associated with the macula retina [18]), *CA9* (zinc metalloenzyme gene), as well as *DCT*, *PMEL*, *MITF*, *TYR*, and *TYRP1* (genes involved in pigment/melanin biosynthesis). This subpopulation also expressed genes involved in early retinal development including of the RPE and eye morphogenesis (*SOX4*, *EFEMP1*, *BMP7*, *VIM*, *GJA1*, and *PTN*) and in the retinoid cycle (*RPE65* and *RLBP1*). In addition, 79 ribosomal genes (32 *RPS* and 47 *RPL*) and 11 mitochondrially-encoded genes were identified. Expression of ribosomal genes and mitochondrially-encoded genes has been correlated with development and maturation [19], including of the retina [20], [21]. This is supported by the GO analysis showing an overrepresentation of pathways involved in mitochondrial and ribosomal functions; protein biogenesis, transport, assembly, and function; as well as ATP biosynthesis and metabolism (Figure 3D; Table S3). Hence, together with the presence of RPE markers, the data indicate this subpopulation comprises a highly metabolically active maturing RPE phenotype.

The subpopulation Common 2 (2204 cells) identified 1077 conserved markers. The 15 most conserved markers in this subpopulation were all known RPE markers. Many of the expressed markers are involved in the generation of RPE cells or in maturation and homeostasis of these cells. For instance, cystatin C encoded by *CST3* is abundantly produced by RPE cells [22] and its secretion diminishes with age [23]. *DCT* is expressed in the developing retina [15] and is important for melanin production and RPE homeostasis [24], [25]. Its downregulation is associated with mature native RPE [26]. This demonstrates that this subpopulation is a mature functional RPE population.

The subpopulation Common 3 (1811 cells) identified 1226 conserved markers. The most conserved markers in this subpopulation were mostly known to be expressed in the RPE (*DCT*, *TTR*, *CST3*, *AQP1*, *FTH1* [27], and *BEST1*), with some markers, such as *TFPI2*, known to promote survival and maintenance of RPE cells [28]. The markers were similar to those of Common 2. Other markers identified are not necessarily RPE-specific, *e.g.*, *GNGT1* that encodes a protein found in rod outer segments, suggesting that cells are not yet fully committed. Together, these markers indicate cellular functions suggestive of functional and maturing RPE cells.

The subpopulation Common 4 (1170 cells) identified 1421 conserved markers. Many of the most conserved markers of this subpopulation are not specifically linked to the RPE. For instance, *TMSB4X* is linked to the cytoplasmic sequestering of NF-κB but has not yet been reported to be associated with molecular events in RPE cells. Many other genes are associated with the cytoskeleton, such as *TAGLN*, *TNNC1*, *CALD1*, *MYLK*, *TPM1*, *ACTA2*, and *MYL9*. On the other hand, there are many markers known to be expressed by the RPE cells, including *TTR*, *BEST1*, *CST3*, *CSTV* [29], *CRYAB*, *SERPINF1*, *PMEL*, *VEGFA*, *RBP1*, *RLBP1*, *TYR*, and *TYRP1*, supporting the RPE identity of this subpopulation. The presence of genes associated with early differentiation, such as *IGFBP5* (downregulated relative to other clusters, as observed upon RPE differentiation [30]) and *CRB2* [31], and genes involved in RPE polarity [32], is indicative of an early stage of RPE maturity and of a differentiating RPE population.

The subpopulation Common 5 (630 cells; 907 conserved markers) was characterised by expression of conserved RPE markers (*SERPINE2* [33], *SFRP5* [34]). Expression of many genes (*AQP1*, *CST3*, *PTGDS*, *SERPINF1*, *BEST1*, and *SMOC2*) was downregulated when compared to that in other populations. Expression patterns of other genes indicate an immaturity/ differentiation or proliferation of cells, such as *GAP43*, *DAAM1*, *CD44* [35], and *DUSP4*. Together, these data describe a subpopulation comprised of cells in early differentiation to RPE.

The subpopulation Common 6 (387 cells; 1664 conserved markers) was characterised by markers associated with retinal cell types other than RPE [36] (such as *SPP1*, *CPODXL*, *STAC2*, and *PCDH9*) or found at low levels in the RPE (such as *CPAMD8* and *SFRP2* [37]). Only the marker *CRABP1* was highly conserved with expression upregulated, whilst expression of other RPE markers (*SERPINF1*, *BEST1*, *RLBP1*, and *RPE65*) was downregulated. These data thus suggest a subpopulation of immature cells.

Finally, the analysis of the PANTHER GO slim biological processes conserved within the Common subpopulations identified pathways that are predominantly involved in mitochondrial, metabolic, and ribosomal processes, as well as purine biosynthesis, nucleotide metabolism, protein biogenesis, localisation, and transport (Figure 3D; Table S3). Altogether, these data demonstrate that the population common to cultures at both time points is heterogeneous, with subpopulations representing different stages of RPE cell differentiation.

### The Young subpopulations are characterised by immature and differentiating cells

Around one third of all cells (5625 cells) were clustered into six Young subpopulations (Table S4). Common RPE markers were conserved within several Young subpopulations (Figure 2A and B; Table S4). These genes were associated with lipid biosynthesis (*PTGDS*), visual cycle (*RGR*, *RLBP1*, *RBP1*, and *BEST1*), melanin biosynthesis (*DCT*, *TYRP1*, *TYR*, *TTR*, and *PMEL*), phagocytic activity (*GULP1*), secretion (*ENPP2, VEGFA*, and *SERPINF1*), and extracellular structure organisation (*ITGAV*, *CRISPLD1*, *CST3*, *EFEMP1*, and *ITGB8*).

The subpopulation Young 0 (2219 cells; 20 conserved markers) comprises singleton cells that expressed *SERPINF1*, *CST3*, *DCT*, *TSC22D4*, *IGFBP5*, and *RNASE1*. Expression of most of these genes has been reported in the retina (Courtesy of Human Protein Atlas, www.proteinatlas.org) [36] and in the human RPE (*IGFBP5* [30]). PANTHER GO biological process analysis indicates involvement of these genes in regulation of neuroblast proliferation, indicative of progenitor cells (Table S4).

The subpopulation Young 1 (1873 cells; 58 conserved markers) was characterised by the expression of *CTNNB1*, *NOG*, *ATP1B1*, *GSTP1*, *CD63*, and *HNRNPH1*. All these genes play fundamental roles in the homeostasis and functions of the RPE. *ATP1B1* encodes an apical Na^+^/K^+^ ATPase, whose expression reduces with age and in age-related macular degeneration (AMD) [38]. Defects in *CTNNB1* are linked to abnormalities in RPE development and pigmentation [39], whereas *GSTP1* is a survival factor for RPE cells, whose expression increases as cells mature [40]; *CD63* is a late endosome/exosome marker known to be released by RPE cells [41]; and *HNRNPH1* levels are associated with improved survival of RPE cells in culture [42]. Hence, expression of these markers indicates functional RPE cells. It is interesting to note that known canonical RPE markers, such as *SERPINF1*, *RLBP1*, *TTR*, *PMEL*, and *CRYAB*, were expressed at lower levels in this subpopulation than in all other subpopulations. GO biological process analysis highlights that this population is highly metabolically active (Table S4). These data are suggestive of an earlier stage of maturation of the RPE cells.

The subpopulation Young 2 (144 cells; 16 conserved markers) was characterised by the specific upregulation of genes including *CCL2*, *SFRP1*, and *B2M*, downregulation of *CTSV* and *TMSB4X*, as well as expression of *DCT*, which is known to be expressed during RPE development [43], indicative of an immature RPE population.

The subpopulation Young 3 (67 cells; 369 conserved markers) was characterised by the expression of genes such as *TOP2A*, *PCLAF*, *PTTG1*, *ANLN*, *MKI67*, *RRM2*, *TPX2*, and *PBK*. Although all genes are found to be expressed in the retina [36], none are associated with a specific RPE signature. However, these genes are associated with cell proliferation (*TOP2A*, *PCLAF*, *PTTG1*, *MKI67*, *RRM2*, *TPX2*, and *PBK)* and cellular rearrangements (*ANLN*), which have been described as characteristics of immature RPE cells [44]. In particular, *ANLN* is reported to promote maturity of intercellular adhesions (tight junctions and adherens junctions) in epithelial cells [45]. TOP2A is associated with retinal development and proliferation [46], which, combined with expression of *PCLAF, PTTG1*, and *MKI67*, suggests a proliferating cell population. Low expression of RPE markers (*RBP1*, *ENPP2*, *CRABP1*, and *HNRNPH1*) further demonstrates the RPE identity of the developing retinal cell subpopulation. Altogether, this expression profile specifies an immature differentiating cell population.

The subpopulation Young 4 (871 cells; 49 conserved markers) was characterised by expression of genes that are not traditionally associated with the RPE identity. The expression of *CRYAB* (downregulated), *CRX*, *FTH1*, *TFPI2* (known to promote survival and maintenance of RPE cells [28]), and *DCT* (expressed in the native RPE) suggests a differentiation to RPE, yet the presence of the photoreceptor-specific *GNGT1* expression could also indicate an early differentiation step where cells are not yet fully committed.

Interestingly, the subpopulation Young 5 (584 cells; 99 conserved markers) displayed a signature comprising mitochondrial and ribosomal transcripts with 9 mitochondrial genes (MT-) and 14 ribosomal genes (RPS- or RPL-). These genes are ubiquitous and have not been specifically correlated to the retina or the RPE, however they are known to facilitate fundamental biological processes, including electron transfer, energy provision, ribosome biogenesis, and protein synthesis. PANTHER GO-slim biological process and GO biological process analyses confirmed that the conserved pathways within this subpopulation are mostly related to the mitochondrial energetic metabolism and nucleotide metabolism (Table S4). This subpopulation also expressed RPE markers, such as *BEST1*, *VEGFA*, *ENPP2*, *TIMP3*, and *TYRP1*, as well as genes involved in early retinal development including of the RPE and eye morphogenesis (*SOX11*, *PMEL*, *EFEMP1*, *BMP7*, *VIM*, *GJA1*, and *PTN*) [15], [47]. Hence the presence of high level of expression of mitochondrial genes and ribosomal genes in RPE is likely significative of highly active cells with high levels of protein synthesis, implying that this is a maturing RPE population.

Taken together, our results demonstrate that all Young subpopulations are immature cells developing to RPE cells.

### The Aged subpopulations are characterised by higher maturity of RPE cells

Less than 10% of all cells (2334 cells) are clustered into the “Aged” category, which comprises six subpopulations (Table S5). All six identified “Aged” subpopulations were subjected to the same analysis as the “Young” and “Common” subpopulations. However, a statistical overrepresentation test returned no positive results for subpopulations Aged 0, 1, and 5, most likely owing to the low number of conserved genes identified. Only a few common RPE markers associated with lipid biosynthesis (*INPP5K*), visual cycle (*RLBP1*), melanin biosynthesis (*TTR* and *DCT*), secretion (*SERPINF1* and *VEGFA*), and extracellular structure organisation (*CST3* and *CRISPLD1*) were conserved in some of the “Aged” subpopulations (Figure 2; Table S5).

The subpopulation Aged 0 (851 cells; 6 conserved markers) is composed of singleton cells. These cells expressed *CRISPLD1*, *PCCA*, *WFDC1*, *TTR*, *SH3BGRL3*, and *TMSB4X*, but at lower average levels per cell than those in all other subpopulations. Some of these genes have been found to be expressed in the RPE (*CRISPLD1*, *WFDC1* [48], *TTR*, and *TMSB4X* [43]) and are associated with late RPE development (*CRISPLD1* and *TTR* [15]). Some other genes have a wider expression pattern (*PCCA* and *SH3BGRL3*) and encode for proteins involved in more universal cellular events. For instance, mitochondrial protein PCCA plays roles in death/survival, SH3BGRL3 is involved in oxidoreduction, whilst *TMSB4X* encodes proteins of the cytoskeleton. Likewise, the subpopulation Aged 1 (815 cells; 12 conserved markers) presented more cells expressing *HSD17B2*, *TPM1*, *MYL9*, *NDUFA4L2*, *BNIP3*, *CALD1*, *TTR*, *DCT*, *MT-CYB*, *FTH1*, *CRYAB*, and *TMSB4X* but at lower average levels per cell than in all other subpopulations*.* Nine out of the twelve genes are known to be expressed in the RPE (*TTR*, *TMSB4X* [43], *CALD1* [49], *DCT* [43], *HSD17B2* [50], *NDUFA4L2* [15], *BNIP3* [27], *FTH1* [27], and *CRYAB*). Some of these markers are associated with late RPE development (*TTR* [15]), whilst others are associated with a geographic localisation of cells within the retina (*HSD17B2* [51] and *NDUFA4L2* [18]). *BNIP3* and *NDUFA4L2* encode for mitochondrial proteins playing roles in death/survival and electron transport, respectively, whilst *TPM1*, *MYL9*, *TMSB4X*, and *CALD1* encode proteins of the cytoskeleton. A similar pattern of expression was observed in the subpopulation Aged 5 (170 cells; 6 conserved markers) with *PCCA*, *WFDC1*, *SERPINE2*, *TMSB4X*, and *TTR* being expressed in more cells with lower average levels than all other subpopulations. In addition, *SPON2*, which encodes ECM proteins important for cell adhesion, was expressed in more cells but at higher levels. The close similarities of these three Aged subpopulations point to a late RPE phenotype, with higher levels of maturation, towards regionalisation of cells.

Similarly, the subpopulations Aged 2 (46 cells; 22 conserved markers), Aged 3 (32 cells; 121 conserved markers), and Aged 4 (420 cells; 34 conserved markers) showed close similarities in terms of gene expression profiles. The presence of known RPE markers, such as *DCT* (Aged 2, downregulated), *CALD1* (Aged 2, downregulated), *TTR* (Aged 3, downregulated; Aged 4), *SOX9* (Aged 3, downregulated), *RBP1* (Aged 3, downregulated; Aged 4), *SERPINF1* (Aged 3, downregulated), *VEGFA* (Aged 4, downregulated), *TMSB4X* (downregulated in all three subpopulations), and *CRYAB* (downregulated in Aged 2 and Aged 3) confirms their RPE identity.

**Figure 4:**
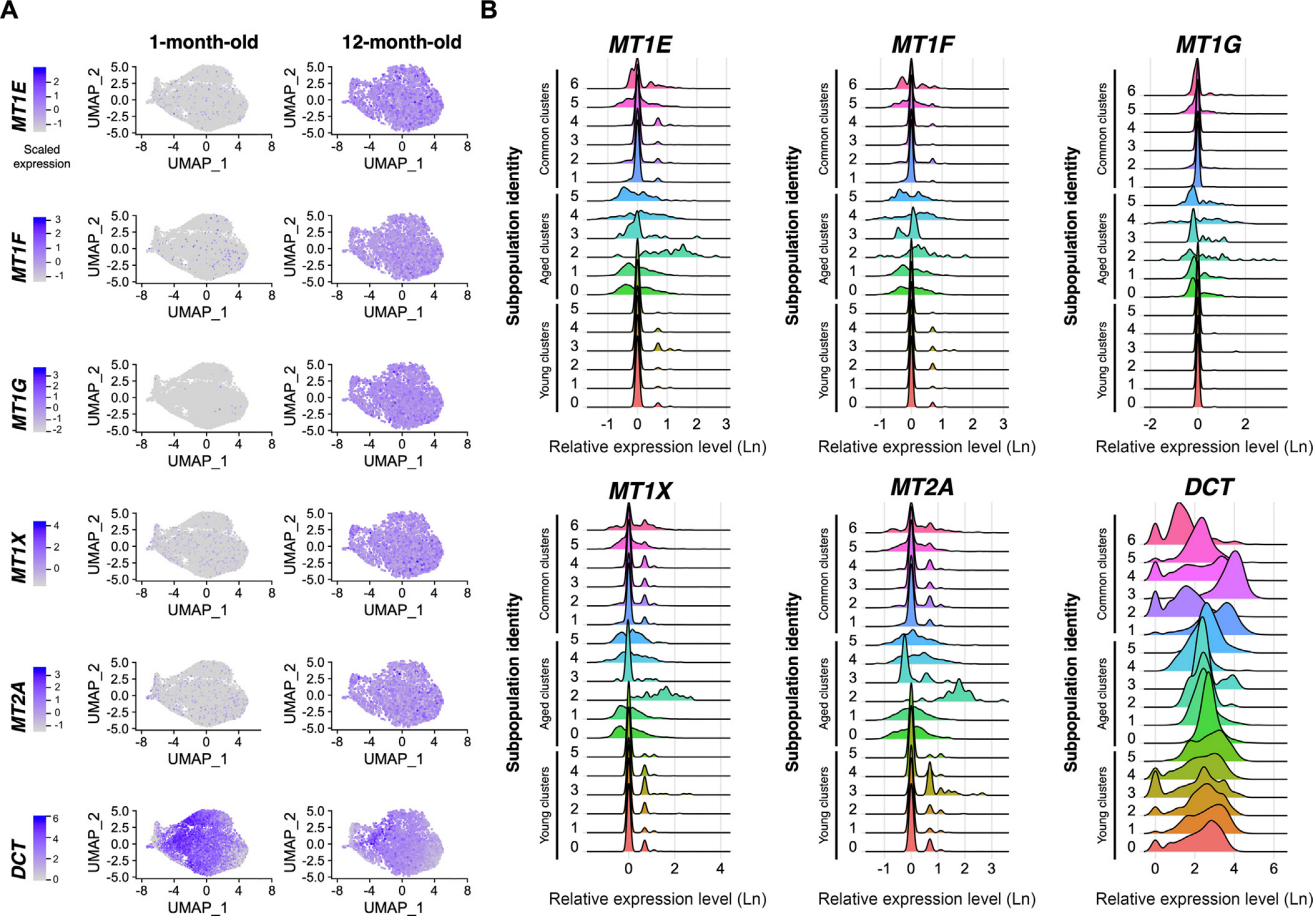
**Aged RPE subpopulations likely increase their handling of metals and antioxidant abilities** **A.** Feature plots of expression profiles of *DCT* and genes encoding key metallothioneins (including *MT1E*, *MT1F*, *MT1G*, *MT1X*, and *MT2A*) across the 1-month-old and 12-month-old cells. The intensity of gene expression is indicated by colour gradient. **B.** Ridge plots of expression profiles (measured in natural log-normalised UMI counts) of *DCT* and genes encoding key metallothioneins and across all subpopulations. Different colours are used for each subpopulation for ease of reading.

PANTHER GO-slim biological process (Aged 2) and PANTHER GO biological process analyses (Aged 2, 3, and 4) were performed. Subpopulations Aged 2 and 4 were similar, with high significance in pathways associated with response to metal ions (particularly cadmium, copper, iron, and zinc); response to stress, chemicals, and toxins; and neural crest fate commitment. Among them, neural crest fate commitment is the most significantly identified biological process in Aged 3, with a 92.9-fold enrichment. Interestingly, GO analysis indicates that nuclear genes encoding metallothioneins involved in metal binding (*MT1E*, *MT1F*, *MT1G*, *DCT*, *MT2A*), as well as in oxidoreduction (*DCT*), were significantly differentially expressed between the “Aged” subpopulations compared to all others, with an overall increased expression per cell as cells age (Table S5). Further analysis of the metallothioneins *MT1E*, *MT1F*, *MT1G*, *MT1X*, and *MT2A* across all subpopulations at the two time points confirms a large increase in their mRNA expression in the 12-month-old culture when compared to the 1-month-old cells (Figure 4A and B). On the other hand, expression of *DCT* was reduced in the 12-month-old culture compared to the 1-month-old cells, which is consistent with a mature RPE profile [26] (Figure 4A and B). Altogether, these data demonstrate that the RPE cells of these “Aged” subpopulations are increasing their handling of metals and antioxidant abilities, which likely reflects a further maturation of the RPE cells.

### RPE cells collected from different time points share common developmental trajectories

**Figure 5:**
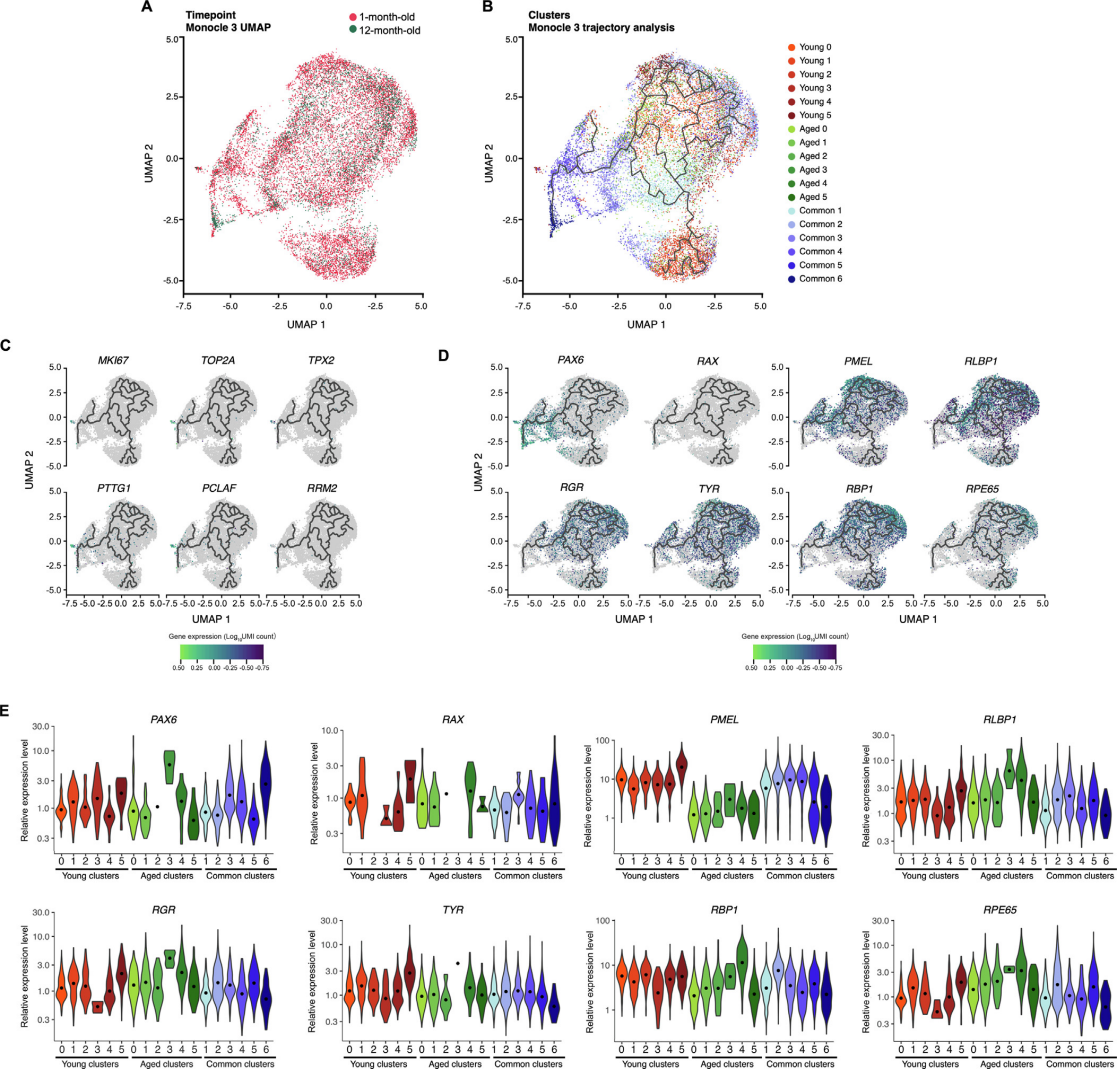
**Only a few cells retain a proliferative profile** Highly dimensional expression data were reduced into two dimensions using UMAP. Plot axes (UMAP1 and UMAP2) represent coordinates in the resulting 2D space. **A.** UMAP of single cell expression profile split by conditions (1-month-old and 12-month-old). **B.** UMAP of single cell expression profile for trajectory analysis using Monocle 3. **C.** UMAP of single cell expression profile for markers associated with proliferation (*MKI67*, *TOP2A*, *PCLAF*, *RRM2*, *TPX2*, and *PTTG1*) across all cells. Expression levels measured as Log_10_ normalised UMI counts are represented by colour intensity. Lines represent differentiation trajectories as calculated by Monocle3. **D.** Pseudotime analysis of early retinal markers (*PAX6* and *RAX*) and RPE genes (*PMEL*, *RLBP1*, *RGR*, *TYR*, *RBP1*, and *RPE65*) across the 1-month-old and 12-month-old cells. Expression levels measured as Log_10_ normalised UMI counts are represented by colour intensity. Lines represent differentiation trajectories as calculated by Monocle3. **E.** Violin plots of normalised UMI counts of early retinal markers and RPE genes across all subpopulations showing variations in expression across cluster groups.

We investigated the pseudo-temporal transition of 1-month-old cells to 12-month-old cells using trajectory analysis methods to identify trajectories originating from proliferative cells in the subpopulation Young 3. *Monocle* 3 [52] identified a complex, branched development trajectory that included cells from both time points (Figure 5A and B). Interestingly, the pseudo-temporal ordering of cells across the trajectory did not correspond to time points (Figure S3A). To determine the nature of the trajectories, we used Moran’s I test to identify 158 genes that were significantly associated with pseudotime (Table S6). These genes formed four co-expression modules that were further analysed with STRING (Table S6; Figure S3B–F). As illustrated in Figure S3, Module 1 revealed genes involved in development (red) and Module 4 revealed genes involved in cell cycle (blue) and mitosis (red). The other two modules did not show clear biological process associations but genes were associated with biological process for neural development. The differential expression of the residual gene trajectory for the four modules confirms differences among various clusters. In particular, all Young subpopulations except Young 4 showed the highest expression levels of genes of the proliferative Module 4 (Figure S3B), confirming their immature proliferative nature.

Differentiated RPE cells are post-mitotic and fully committed to the RPE lineage, in large part due to their cell–cell contact inhibition [53], [54]. The expression of the proliferation marker *MKI67* and of other genes associated with proliferation (*TOP2A*, *TPX2*, *PTTG1*, *PCLAF*, and *RRM2*) was examined to assess whether some progenitor populations remain with proliferative potentials, and whether these vary over time in culture. Our data indicate that only a small portion of cells remained proliferative over time in culture. In particular, *MKI67* was almost uniquely expressed in the immature subpopulation Young 3, together with other cell proliferation markers (*TOP2A*, *TPX2*, *PTTG1*, *PCLAF*, and *RRM2*; Figure 5C; Table S4). Very few cells of the 12-month-old population were found to express markers associated with proliferation (Figure 5C). This confirms that only a small subpopulation of progenitor/immature cells present early in culture is proliferative and these cells disappear with time in culture.

Pseudotime analyses of early retinal markers (*PAX6* and *RAX*) and RPE genes (*PMEL*, *RLPB1*, *RGR*, *TYR*, *RBP1*, and *RPE65*) were performed in all subpopulations to measure gene expression trajectories (Figure 5D and E; Figure S4). The early retinal markers *PAX* and *RAX,* were expressed in similar manners across subpopulations, with minor variations between samples, with the exception of the subpopulation Aged 3, which showed higher levels of *PAX6* and absence of expression of *RAX* (Figure 5D and E; Figure S4). The pigmentation marker *TYR* was consistently expressed across all populations, whilst expression of *PMEL* was downregulated in all aged subpopulations and in subpopulations Common 5 and 6 (Figure 5D and E; Figure S4). Similarly, comparable expression levels of *RGR*, *RLBP1*, and *RBP1* were observed across all subpopulations, with the exception of subpopulations Aged 3 and 4, which showed higher levels of expression (Figure 5D and E; Figure S4). These variations in gene expression are likely indicative of RPE cell maturation in culture.

### RPE subpopulations contribute to paracrine signalling

**Figure 6:**
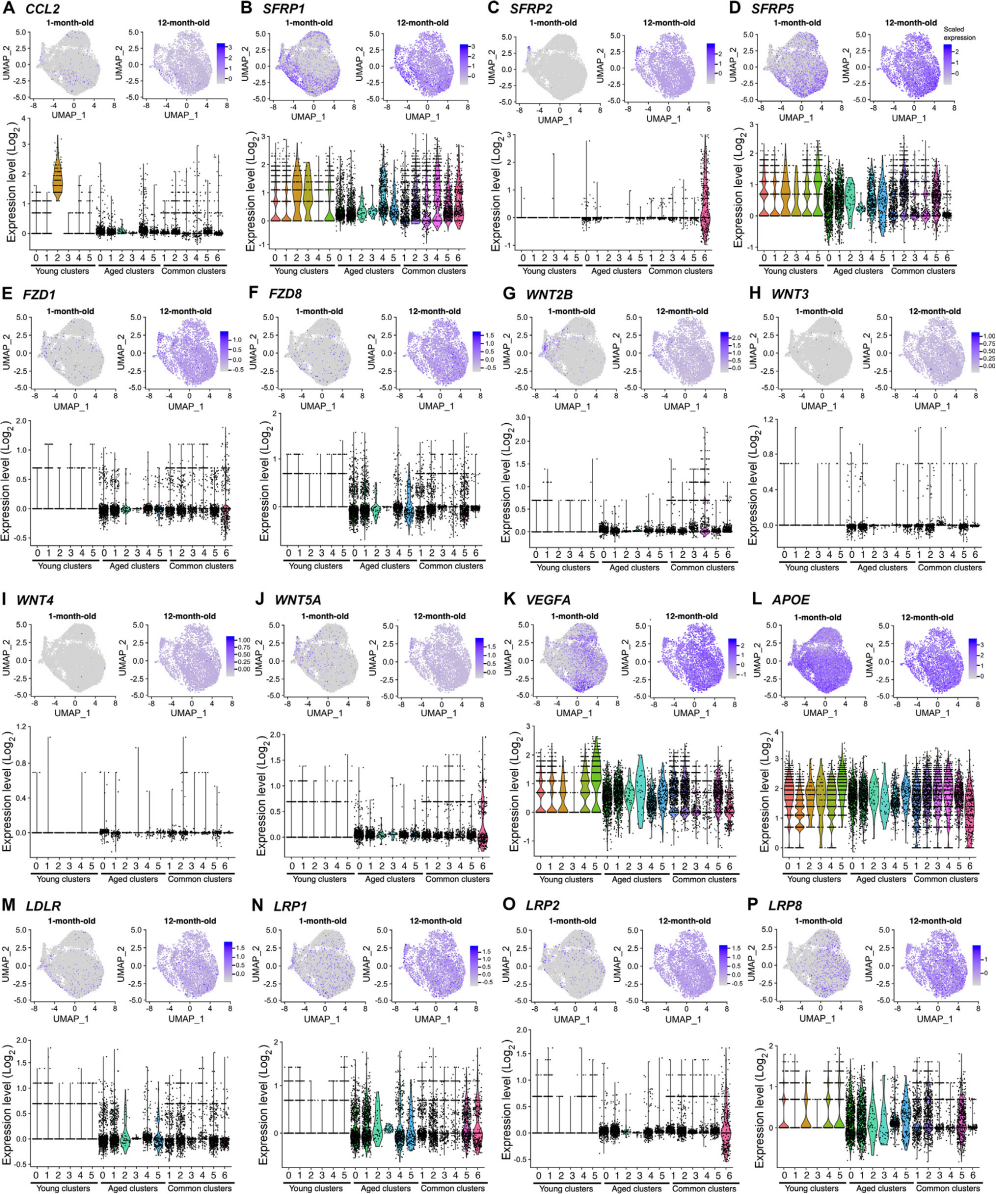
**RPE subpopulations contribute to paracrine signalling** UMAP of single-cell expression profile of markers for 1-month-old and 12-month-old cultures, with associated violin plots, representing Log_2_ UMI counts across all populations for *CCL2* (**A**); *SFRPs*, *FZDs*, and *WNTS* (**B**); *VEGFA* (**C**); *APOE*, *LDLR*, and *LRPs* (**D**). In the UMAPs, the intensity of gene expression is indicated by colour gradient. UMAP of single-cell expression profile of markers for 1-month-old and 12-month-old cultures, with associated violin plots across all populations for *CCL2* (A), *SFRP1* (B), *SFRP2* (C), *SFRP5* (D), *FZD1* (**E**), *FZD8* (**F**), *WNT2B* (**G**), *WNT3* (**H**), *WNT4* (**I**), *WNT5A* (**J**), *VEGFA* (**K**), *APOE* (**L**), *LDLR* (**M**), *LRP1* (**N**), *LRP2* (**O**), *LRP8* (**P**). In the UMAP plots, the intensity of gene expression is indicated by colour gradient. In the violin plots, different colours are used for each subpopulation for ease of reading.

RPE cells can modulate immune responses and differentiation of other retinal cell types, in part via paracrine signalling [1]. Such modulation can be detected *in vitro* through examining expression of specific secretion factors and receptors known to play roles in immune or developmental events. For instance, in the retina, the chemokine CCL2 is implicated in monocyte infiltration following damage [55] and its secretion by RPE cells contributes to the regulation of the immunological response to inflammation [56]. *CCL2* was found to be faintly expressed in cells at both time points (Figure 6A). *CCL2* was identified as a marker of the immature subpopulation Young 2 (Table S2) and was also expressed, albeit at lower levels, across other “Aged” and “Common” subpopulations (Figure 6A). Unsurprisingly, *CCR2*, which encodes the cognate receptor of CCL2, was not expressed in any subpopulations, as it is most likely that CCL2 targets monocytes but not endogenous cells of the retina (data not shown). *CCL2* was more uniformly expressed in the 12-month-old sample, suggesting more homogeneity in its expression as cells mature.

Secreted frizzled-related proteins (SFRPs) are secreted ligands for receptors Frizzled (FZD1–10), which are also receptors for WNT proteins (WNTs). Therefore, SFRPs are considered WNT antagonists and can modulate WNT signalling. Cells in culture from both time points expressed genes involved in the WNT signalling pathways, in particular *SFRP 1*, *2*, and *5*; *FZD 1* and *8*; as well as *WNT 2B, 3*, *4*, and *5A*, with more expression homogeneity in the 12-month-old samples for many of them (Figure 6B–J). *SFRP1* was expressed across all subpopulations with varied levels and was identified as a marker of the immature subpopulation Young 2 (Figure 6B; Table S4). *SFRP2* was identified as a marker of the immature subpopulation Common 6 where it was mainly expressed, whilst *SFRP5* was identified as a marker of the immature subpopulation Common 5 and expressed across most subpopulations (Figure 6C and D; Table S3). It is interesting to note that expression of *SFRP1* and *SFRP5* was both downregulated in the subpopulation Aged 3 (relative to other “Aged” subpopulations), reflective of a dynamic expression pattern of *SFRP* genes by RPE cells in culture (Figure 6C and D; Table S3). Interestingly, 1-month-old RPE cells showed low expression levels of *FZD1*, *FZD8*, *WNT2B*, *WNT3*, *WNT4*, and *WNT5A* with expression of all other *FZD* and *WNT* genes undetectable (Figure 6E–J). The expression of all detectible *SFRP*, *FZD*, and *WNT* genes was higher in the 12-month-old RPE cells (Figure 6B–J). This dynamic profile of expression of molecules involved in WNT signalling indicates that RPE cells can signal via the WNT pathway. This correlates with the known role of WNT signalling in retinal development including the RPE [34], [57].

Pigment epithelium-derived factor (PEDF) is another factor key to various retinal cell differentiation, maturation, and survival, including RPE and photoreceptors [58]. It is encoded by *SERPINF1*, which is highly expressed by RPE cells and was found as a marker of subpopulations within the Young, Common, and Aged subpopulations (Figure 1D; Tables S3–S5). However, the *PNPLA2*, the gene encoding PEDF receptor, was not found to be expressed in the RPE cells, indicating that PEDF secreted from RPE largely acts in a paracrine manner. Likewise, *VEGFA*, the gene encoding pleiotropic factor VEGFα [59], was also identified as a marker of subpopulations within the three populations, but no expression of the gene encoding VEGF receptor (*VEGFR1*–*3*) was detected in the samples, again indicating that the RPE-secreted VEGF likely acts on neighbouring cells to maintain homeostasis (Figure 6K; Tables S3–S5). These examples of paracrine molecules secreted by RPE subpopulations illustrate the important role played by RPE cells in the shaping of a developing retina. These cells may release bioactive factors to regulate events such as cell fate, differentiation, polarity, and maturity, and contribute to the regulation of the immune environment of the retina.

### *APOE* is a conserved marker of various RPE subpopulations

Apolipoproteins E (APOE) are proteins involved in lipid metabolism including cholesterol and are also regulated with complement activation in the RPE [60]. APOEs interact with the low-density lipoprotein (LDL) receptors (LDLRs) and very-low-density lipoprotein receptors (VLDLRs). *APOE* was highly expressed in cells across both time points in culture (Figure 6L). *APOE* was identified as a conserved marker of the subpopulations Young 1 and 5, as well as all Common subpopulations (Figure 6L; Tables S3 and S4). The levels of *APOE* expression per cell was however different between subpopulations, with an upregulation in Young 5, and a downregulation in Young 1 as well as in Common 1, 2, 3, and 6 (Figure 6L). Interestingly, within the subpopulation Common 4, cells arising from the 1-month-old culture expressed lower levels of *APOE* than cells arising from 1-month-old culture of all other Common subpopulations, whereas the opposite was observed for cells from 12-month-old culture (Table S3). The opposite pattern was observed in the subpopulation Common 5 (Table S3). Expression of *LDLR*, *LRP1*, *LRP2*, and *LRP8*, the genes encoding APOE receptors, was detected in both cell cultures, with increased levels in the 12-month-old samples when compared to the 1-month-old culture (Figure 6M–P). Interestingly, expression of these receptor genes was basically absent from all Young subpopulations, but present at higher levels in the Aged subpopulations (Figure 6M–P). These data illustrate that the expression of *APOE* and associated receptor genes is dynamic within RPE subpopulations and with time in culture.

### The complement pathway is not modulated with time in culture

RPE cells express many complement components in various retinal diseases, inflammation, and/or ageing [61]. *C1s*, *C1r*, and *C1QBP*, the genes encoding complement regulators that form the C1 complex, were conserved markers in a few Young and Common subpopulations (Tables S3 and S4). Other genes associated with the complement response (such as *CFH*, *CFB*, *CFHR1*, *CFHR3*, and *C3*) were not identified as markers of any subpopulation (Tables S3–S5). This suggests that the complement components are not modulated with time in culture.

### RPE cells do not undergo epithelial mesenchymal transition

It is interesting to note that no markers of epithelial mesenchymal transition (such as *SNAI1*, *SNAI2*, *ZEB1*, *TWIST1*, and *GSC*) were characterised in any examined subpopulation (Tables S3–S5). These data demonstrate the stability of the cell culture over time, with no evidence of a transition to a mesenchymal phenotype.

### RPE subpopulations express native RPE markers with different patterns

**Figure 7:**
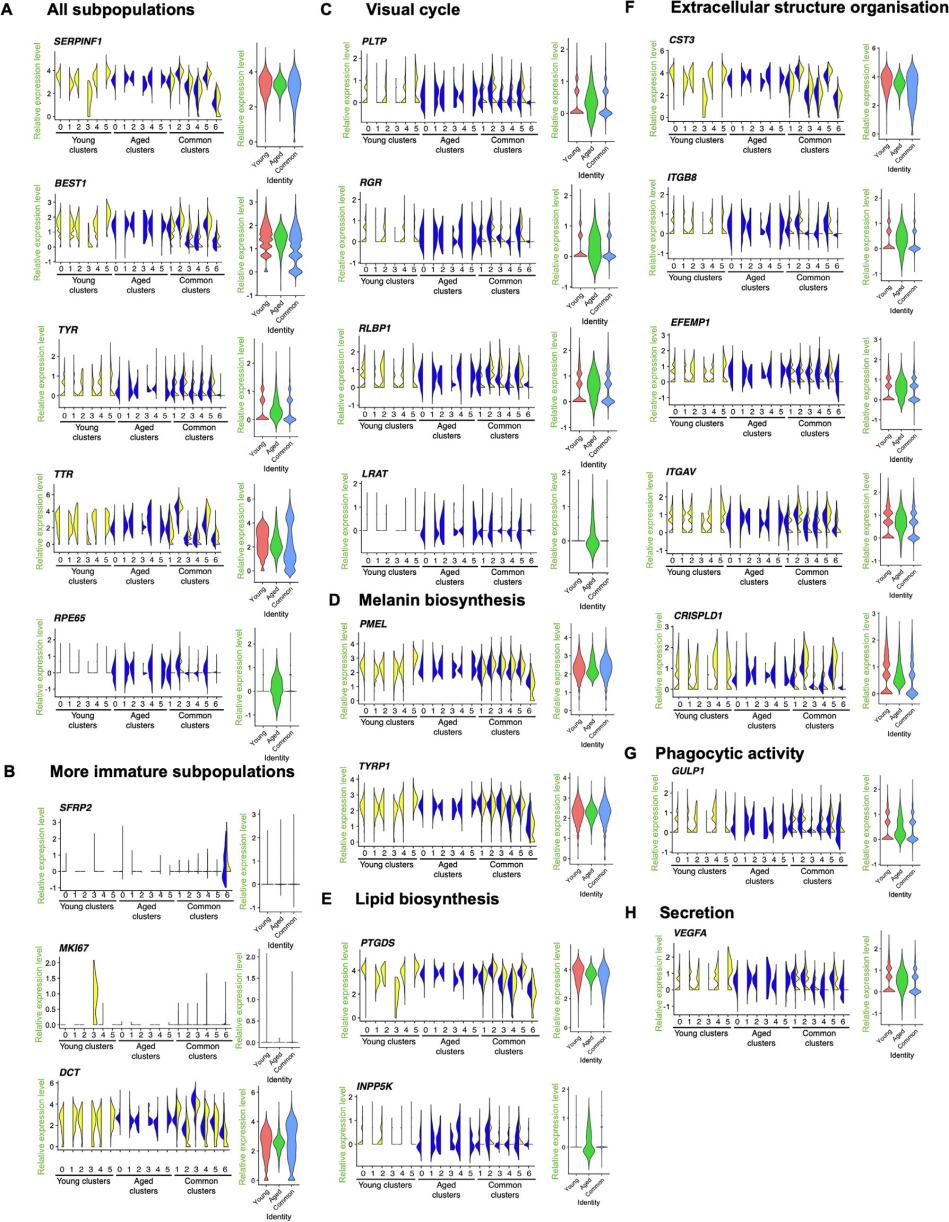
**hPSC-derived RPE subpopulations express native RPE markers with different patterns** Violin plots of selected markers representative of native RPE cells (obtained from [28]) across all subpopulations and in the three main populations “Young”, “Aged”, “Common”, represented in different colours. Subpopulations arising from the 1-month-old culture are indicated in yellow; subpopulations arising from the 12-monht-old culture are indicated in blue. **A.** Genes found in all subpopulations (*SERPINF1*, *BEST1*, *TYR*, *TTR*, and *RPE65*). **B.** Genes found in more immature subpopulations (*SFRP2*, *MKI67*, and *DCT*). **C.** Genes with varied expression associated with visual cycle (*PLTP, RGR, RLBP1*, and *LRAT*). **D**. Genes with varied expression associated with melanin biosynthesis (*PMEL* and *TYRP1*). **E.** Genes with varied expression associated with lipid biosynthesis (*PTGDS* and *INPP5K*). **F**. Genes with varied expression associated with extracellular structure organisation (*CST3*, *ITGB8*, *EFEMP1*, *ITGAV*, and *CRISPLD1*). **G**. Genes with varied expression associated with phagocytic activity (*GULP1*). **H.** Genes with varied expression associated with secretion (*VEGFA*). The plots describe the distribution and relative expression of each gene in the subpopulations, measured as normalised UMI counts.

**Figure 8:**
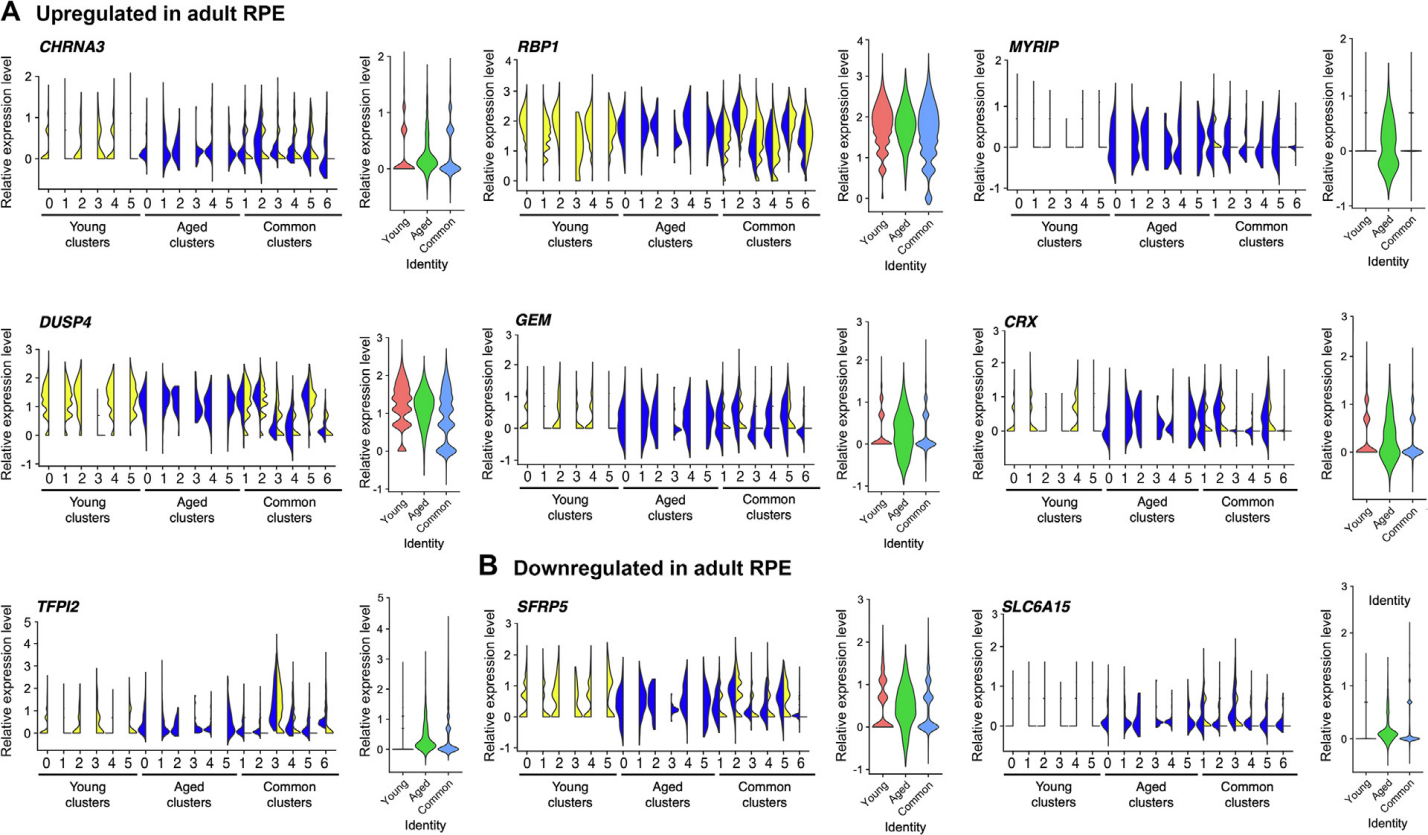
**Some hPSC-derived RPE subpopulations acquire a gene expression profile closer to adult native RPE with time in culture** Violin plots of selected markers representative of native adult RPE cells (obtained from [28]) across all subpopulations and in the three main populations “Young”, “Aged”, “Common”, represented in different colours. Subpopulations arising from the 1-month-old culture are indicated in yellow; subpopulations arising from the 12-monht-old culture are indicated in blue. **A.** Genes with upregulated expression in adult RPE (*CHRNA3, RBP1*, *MYRIP*, *DUSP4, GEM, CRX*, and *TFPI2*). **B.** Genes with downregulated expression in adult RPE (*SFRP5* and *SLC6A15*). The plots describe the distribution and relative expression of each gene in the subpopulations, measured as normalised UMI counts.

To assess correspondence of the RPE cells with native counterparts, we compared the hPSC-derived RPE signature to that of foetal native RPE cells, which was examined using scRNA-seq previously [15]. As observed in foetal native tissue, most subpopulations of hPSC-derived RPE cells expressed *SERPINF1*, *BEST1*, *TYR*, *TTR*, and *RPE65*, and the more immature subpopulations also expressed *MKI67* and *DCT* (Figure 7A and B). Genes commonly expressed in native RPE cells were enriched in 12-month-old subpopulations and in Aged subpopulations (such as *RPE65*, *LRAT*, *PLTP*, *RGR*, *RLBP1*, *LRAT*, *INPP5K*, *ITGB8*, *EFEMP1*, *ITGAV*, *GULP1*, and *VEGFA*), whilst other genes, including *TYRP1*, were found at similar expression levels in 1-month-old and 12-month-old cultures, as well as in Young, Common, and Aged populations (Figure 7C–H). In addition, expression of some genes, such as *PMEL*, *PTGDS*, *CST3*, and *CRISPLD1*, was significantly lower in 12-month-old cultures than that in 1-month-old cultures (Figure 7D–F). Some common RPE genes are expressed in the adult native RPE at much higher levels than in foetal RPE [26]. These include *TTR*, *RPE65*, *BEST1*, *CHRNA3*, *RBP1*, *MYRIP*, *TFPI2*, *PTGDS*, *SERPINF1*, *DUSP4*, *GEM*, and *CRX*. Similarly, downregulation of *DCT*, *SFRP5*, *TYRP1*, and *SLC6A15* is associated with mature native RPE [26]. We thus compared the expression profiles of these genes in the RPE cultures over time, in order to assess the maturity of the cultured cells [26] (Figure 8). *CHRNA3*, *MYRIP*, *GEM*, *CRX*, and *TFPI2* — whose expression is upregulated in the adult native RPE— were more highly expressed in the Aged population (Figure 8A). However, *PTGDS*, *RBP1*, and *DUSP4*, which are genes also highly expressed in the adult RPE, were not found at higher levels in the Aged or 12-month-old cultures than in the Young population or 1-month-old culture (Figures 7E and 8A). Expression of *DCT*, *PMEL*, *TYRP1*, *SFRP5*, and *SLC6A15* was generally downregulated in the Aged subpopulations and in the 12-month-old Common subpopulations, when compared to the 1-month-old Common subpopulations (Figures 7B and D; 8B).

## Discussion

Here, we provide a dynamic profile of the transcriptome of hPSC-derived RPE cells over 12 months. Our data confirm expression of marker genes of RPE homeostasis and functions in hESC-derived RPE [15], [26], [62], [63] and provide novel information on the timing of expression of these markers. At both early and late time points, we observed that hPSC-derived RPE cells expressed genes associated with lipid metabolism, secretion, visual cycle, melanin synthesis, phagocytic activity, metal binding, and oxidoreductase activity. Based on expression pattern of genes associated with RPE maturity levels and on PANTHER GO analyses, the 18 subpopulations identified regroup into populations from immature and progenitor cells, to maturing RPE cells, and functionally mature RPE cells (based on genes involved in RPE functions). Some subpopulations comprised highly metabolically active cells. The pseudo-temporal analysis could not identify trajectories matching time points, which is likely due to the experimental design of including two time points only. Beyond the scope of this study but of interest, including intermediate culture time points could provide more information on the pseudo-temporal ordering of cells alongside the course of culture.

An essential function of the RPE is photoprotection of the retina, which is accomplished through different mechanisms. These include absorbing radiation, binding and sequestering redox-active metals such as iron, as well as scavenging free radicals and reactive oxygen species [64]. Metallothioneins are metal-binding proteins that are protective against oxidative stress. Compared to the 12-month-old RPE cells, the 1-month-old RPE cells express fewer transcripts for the metallothioneins *MT1E*, *MT1F*, *MT1G*, *MT2A*, and *MT1X*, as well as higher levels of *DCT*. These data suggest a variation in the handling of metals and in the antioxidant abilities of RPE cells, which could be reflective of either a necessity to handle more oxidative stress in an *in vitro* ageing environment or a maturation of RPE cells towards a more mature and protective phenotype [64]. The assessment of variations in the expression of other genes between the two time points indicates a maturation profile of cells rather than an increased stress. Indeed, *DCT* is known to be expressed in human retinal progenitor cells [15] and its expression is regulated by the early RPE marker MITF. It is thus not surprising that as RPE cells mature, *MITF* expression reduces [65] and subsequently *DCT* expression reduces, as is observed in human foetal retina [15]. Similarly, *CTNNB1* regulates *MITF* and *OTX2* expression and subsequently RPE differentiation [39]. Finally, *SOX11* is known to be expressed in early retinal progenitor and early in differentiating RPE cells [15], [47], hence its downregulation as cell culture ages further supports a maturation of RPE cells in culture. The subpopulations Young 0 and Young 1 are characterised by the expression of *CST3*, which encodes the cysteine proteinase inhibitor cystatin. Interestingly, this protein is known to decrease in native RPE cells with ageing [23]. Its presence in the cell population further strengthens the implication of a maturing RPE population over time. The high expression levels of mitochondrial and ribosomal genes in some cell populations likely indicate that cells are metabolically and transcriptionally active, necessitating energy and ribosomal activities for protein synthesis, respectively. It could also suggest that ribosomes potentially contribute to extra-ribosomal functions in the RPE cells, such as cell development and maturation [19], as already reported for melanocyte development [66], retinal development [20], [21], and retinal degeneration [67]. Of note, the absence of markers of epithelial mesenchymal transition highlights the robustness and ability of RPE cells to maintain their phenotype *in vitro* despite of prolonged culture time.

The expression analysis of ligands and receptors involved in retinal development and homeostasis demonstrates a dynamic profile of gene expression of hPSC-derived RPE cells. This highlights the importance of the RPE in the development and homeostasis of the whole retina. For instance, WNT signalling is important to the early stages of RPE differentiation [39], [68]. Some *SFRPs* and *WNTs* are expressed in RPE cell subpopulations and the genes encoding FZD receptors are very lowly expressed in most subpopulations. This suggests that the ligand expression could be directed to paracrine signalling, playing roles in the development and homeostasis of other neighbouring retinal cells. For instance, *SFRP 1*, *2*, and *5*, found to be expressed in the hPSC-derived RPE cells, are associated with retinal development [34], [57], and known to promote the differentiation of retinal ganglion cells and photoreceptors [69], and axon guidance [70]. Similarly, high expression levels of *PEDF/SERPINF1* and of *VEGF* were found across many subpopulations, yet genes encoding their respective receptors, *PNPLA2* and *VEGFR*, were not expressed in the cells, suggestive of a paracrine signalling mechanism between RPE and neighbouring cells as well. This is in accordance with the role of these growth factors in the biology and survival of retinal cells, including photoreceptors [58], [71] and retinal ganglion cells [72], or beyond the retina in neighbouring endothelial cells. Interestingly, *APOE* was also expressed in cells across both time points in culture, with variations observed between subpopulations. In the RPE, APOE is involved in lipid metabolism including cholesterol and drusen content [60]. APOE also plays a striking role in melanogenesis, regulating the formation of functional premelanosome protein (PMEL) amyloid fibrils in RPE cells [73]. Hence, the variations observed in its expression levels and that of its receptors could possibly be indicators of melanogenesis within RPE subpopulations. Altogether, the expression analysis of genes encoding ligands and receptors in RPE cells also hints at the possible value of co-culturing RPE cells with retinal organoid cultures to further support retinal differentiation and cell maturation, and to improve the *in vitro* modelling of retinal biology.

Our analyses also reveal that cells in culture can develop a transcriptomic profile more closely related to the adult native RPE with higher expression levels of some RPE genes and lower expression levels of others, as observed in their native counterpart. Altogether, these data thus strongly suggest that as hPSC-derived RPE cells mature with time in culture, they acquire characteristics more closely resembling those of an adult RPE profile.

## Conclusion

The novel insight into the underlying genetic architecture of hPSC-derived RPE cells at short and long durations in culture conditions reveals a gradual differentiation and maturation process, as well as a stable RPE phenotype over time. Most cells with a clear RPE signature are found in the Common subpopulations, indicating that RPE cells are present from an early time point in culture and maintain this identity with time. The clustering analysis also reveals that whilst some subpopulations express more genes associated with retinal and RPE biology, other RPE subpopulations demonstrate increased expression in mitochondrial and/or ribosomal genes. Altogether, these data suggest that hPSC-derived RPE cells develop their characteristic signatures early during the differentiation process and continue to mature over time in culture. Our analysis also warrants the use of hPSC-derived RPE cells for modelling of RPE biology at early and later differentiation timings.

## Materials and methods

### Cell culture and differentiation of hESCs to RPE cells

The hESC line H9 (Wicell) was maintained on vitronectin-coated plates using StemFlex (Catalogue No. A3349401, ThermoFisher Scientific, Waltham, MA), with medium changed every second day [74]. Cells were differentiated into RPE cells as previously described [3] with the following modifications. Briefly, hESCs were maintained in culture until reaching 70%–80% confluency, at which stage StemFlex was replaced with Complete E6 (Catalogue No. 05946, Stem Cell Technologies, Vancouver, Canada) supplemented with N2 (Catalogue No. 17502048, ThermoFisher Scientific) to induce retinal differentiation, with media changes 3 times/week for 33 days. On Day 33, medium was replaced with RPEM containing α-MEM (Catalogue No. 12571071, ThermoFisher Scientific), 5% foetal bovine serum (Catalogue No. 26140079, Thermo-Fisher Scientific), non-essential amino acids (Catalogue No. 11140050, ThermoFisher Scientific), penicillin- streptomycin- glutamine (Catalogue No. 10378016, ThermoFisher Scientific), N1 (Catalogue No. N6530, Sigma-Aldrich, St Louis, MO), and taurine-hydrocortisone-triiodo-thyronin (in-house) to promote RPE differentiation, with medium changed every second day. Cells were cultured for 32 days, a time point at which maximal pigmentation is routinely observed. Cells were harvested with an 8-min exposure to 0.25% trypsin-EDTA (Catalogue No. 25200056, ThermoFisher Scientific) and inactivated with RPEM. Non-RPE contaminants (visible as unpigmented cells) were manually removed from the culture, which begin shedding off the culture plate after ~2 min. Cells were seeded at a density of 75,000 cells/cm^2^ onto growth factor-reduced Matrigel-coated tissue culture plates (Corning Matrigel hESC-qualified Matrix, Catalogue No. 354277, *In vitro* Technologies, Melbourne, Australia). Media was changed every second day, with the first sample of cells harvested after 30 days (D30) and the second sample harvested on day 367 (D367) for scRNA-seq analysis (Figure 1A).

### RPE cell harvest and single-cell preparation

RPE cells were dissociated to single cells using 0.25% trypsin-EDTA for 8 min and inactivated with RPEM. Cells were centrifuged at 300 g for 1 min to pellet and resuspended in a small volume of RPEM containing 0.1% v/v propidium iodide (PI, Sigma-Aldrich) to exclude non-viable cells. Single cell suspensions were passed through a 35-µm filter prior to sorting. A minimum of 60,000 live cells (PI-negative) were sorted on a BD FACSAria IIu (100 µm, 20 psi; BD-Biosciences, San Jose, CA) into culture medium. Cells were centrifuged at 300 g for 5 min and resuspended in PBS containing 0.04% BSA to a concentration of ~800–1000 cells/µl. Approximately 17,400 cells were loaded onto a 10X chip for a target recovery of 10,000 cells. Cultures at the two time points were captured separately to prepare two separate 10X reactions.

### Generation of single-cell gel beads in emulsion and sequencing libraries

To generate single-cell gel beads in emulsion, single cell suspensions were loaded onto 10X Genomics Single Cell 3′ Chips together with the reverse transcription master mix following the manufacturer's protocol for the Chromium Single Cell 3′ v2 Library (PN-120233, 10X Genomics, Pleasanton, CA). For each sample, sequencing libraries were generated with unique sample indices (SI), assessed by gel electrophoresis (Agilent D1000 ScreenTape Assay, Santa Clara, CA) and quantified with qPCR (Illumina KAPA Library Quantification Kit, Roche, Pleasanton, CA). Following pooling and normalisation to 4 nM, libraries were denatured and diluted to 1.6 pM for loading onto the sequencer. Libraries were sequenced on an Illumina NextSeq 500 (NextSeq Control Software v2.2.0/Real Time Analysis v2.4.11) using NextSeq 500/550 High Output Kit v2.5 (150 Cycles) (Catalogue No. 20024907, Illumina, San Diego, CA) as follows: 26 bp (Read 1), 8 bp (i7 Index), and 98 bp (Read 2).

### Preprocessing, mapping, and quantification of scRNA-seq data

We used the *cellranger mkfastq* and *cellranger count* pipelines from the Cell Ranger Single Cell Software Suite (version 3.0.2) by 10x Genomics (http://10xgenomics.com) for initial quality control, sample demultiplexing, mapping, and quantification of raw sequencing data. The *cellranger count* pipeline was run with the following argument: “--expect-cells = 10,000”, and reads were mapped to the *Homo sapiens* reference genome (GRCh38, Annotation: Gencode v29). Filtered count matrices were then used for downstream analyses in R.

### Quality control and normalisation

**Supplementary figure S1:**
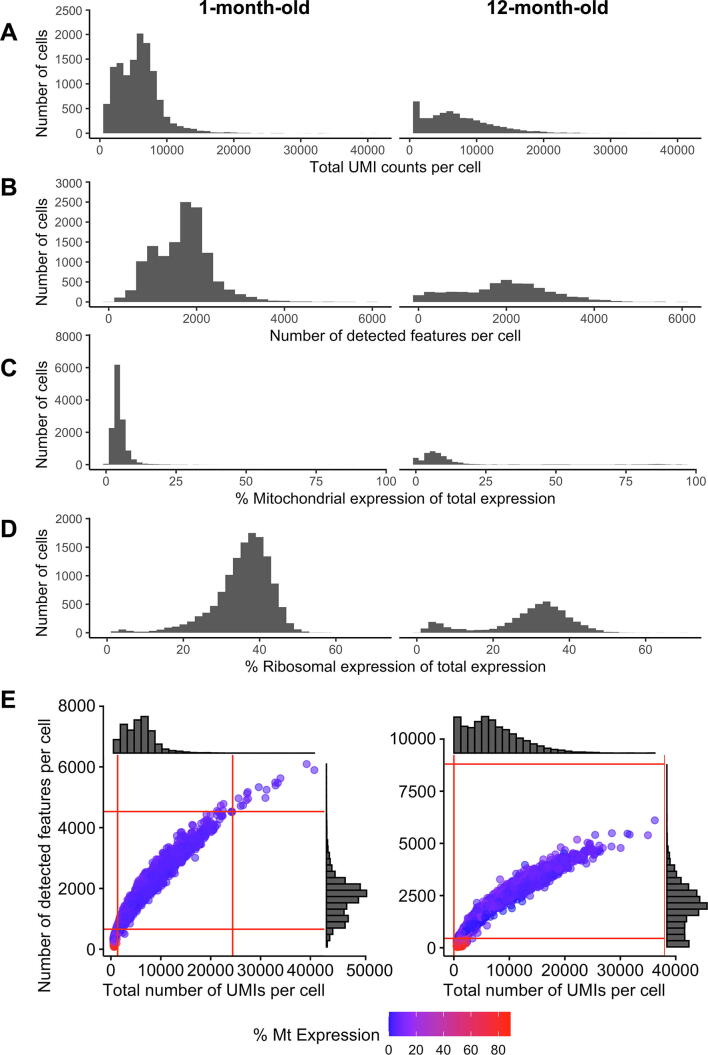
**Filtering of cells based on quality control metrics. A**. Total number of UMIs per cell. **B**. Total number of features per cell. **C**. Percentage of mitochondrial gene expression relative to total expression. **D**. Percentage of ribosomal gene expression relative to total expression. **E**. Relationship between total UMIs, detected genes, and percentage of mitochondrial gene expression. Histograms for total UMIs and detected features are located on X and Y margins of scatter plot, respectively. Scatter plot represents the relationships between the two metrics and are coloured by percentage of mitochondrial expression. Plots for 1-month-old culture and 12-month-old culture are shown on the left and right, respectively. UMI, unique molecular identifier; MAD, median absolute deviation.

Using Seurat v3.1.3 [16], data from the two culture time points underwent quality control and normalisation separately. The following values were calculated for each cell: total number of unique molecular identifiers (UMIs), number of detected genes, as well as proportion of mitochondrial and ribosomal transcripts relative to total expression. Cells were removed from subsequent analysis if the total UMI count of the cell exceeded the threefold median absolute deviation (MAD) across all cells in the sample. Manual thresholds were derived from outlier peaks in the distributions of the number of detected genes, and fraction of mitochondrial and ribosomal transcripts to total expression (Figure S1). Cells were removed from the 1-month time point sample if the number of detected genes exceeded the lower and upper limits of 220 and 5000, respectively, while cells from the 12-month time point sample were removed if the number of detected genes was lower than 220. Cells from both time points were removed if mitochondrial transcripts accounted for more than 25% of total expression, and/or ribosomal transcripts accounted for more than 60% of total expression (Figure S1A–D; Table S1). The confounding effect of these mitochondrial and ribosomal transcript QC metrics in remaining cells was regressed out during cell–cell normalisation, using the *SCTransform* function from Seurat [75] (Table S7).

### Integration, dimensionality reduction, and clustering

**Supplementary figure S2:**
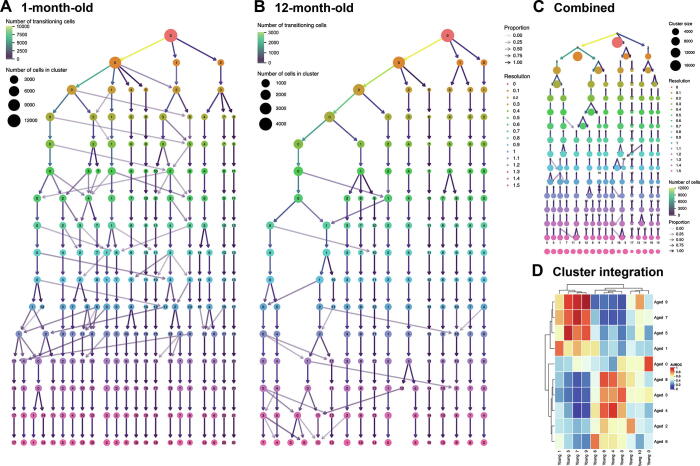
**Characterisation of stable cell subpopulations**. Graph-based clustering was performed at different resolutions ranging from 0 to 1.5, in increments of 0.1 for 1-month-old (**A**), 12-month-old (**B**), and combined (**C**) datasets using clustree. Regions of stability are represented by minimal branching. Values within circles are arbitrary cluster identifiers and arrows indicate the proportion of cells moving from one group to another (transitioning cells, colour coded). **D**. Integration of clusters identified in 1-month-old and 12-month-old cells via MetaNeighbor. Heatmap represents degree of similarity of clusters as AUROC values. AUROC, area under receiver operating characteristic curve.

**Supplementary figure S3:**
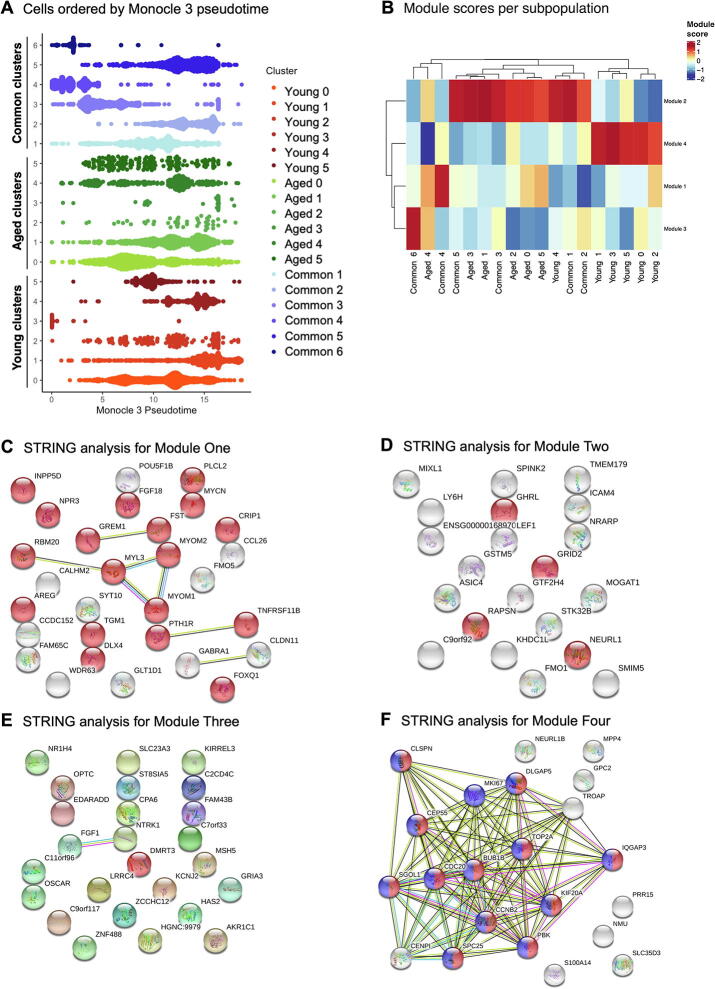
**Residual trajectory genes modules analyses measured with Monocle 3**. **A.** Monocle 3 pseudotime represented in beeswarm plots. Different colours are used for each subpopulation for ease of reading. **B.** Heatmap of module scores for each subpopulation. Modules were identified via Monocle 3 gene trajectory analysis, and module scores were calculated via differential expression of genes from each module. **C**. STRING analysis of genes from Table S6 for Module 1. Genes involved in regulation of development are shown in red. **D**. STRING analysis of genes from Table S6 for Module 2. Genes involved in regulation of synapse organisation are shown in red. **E**. STRING analysis of genes from Table S6 for Module 3. No relevant biological processes was revealed. **F**. STRING analysis of genes from Table S6 for Module 4. Genes involved in regulation of cell cycle and mitosis are shown in blue and red, respectively.

**Supplementary figure S4:**
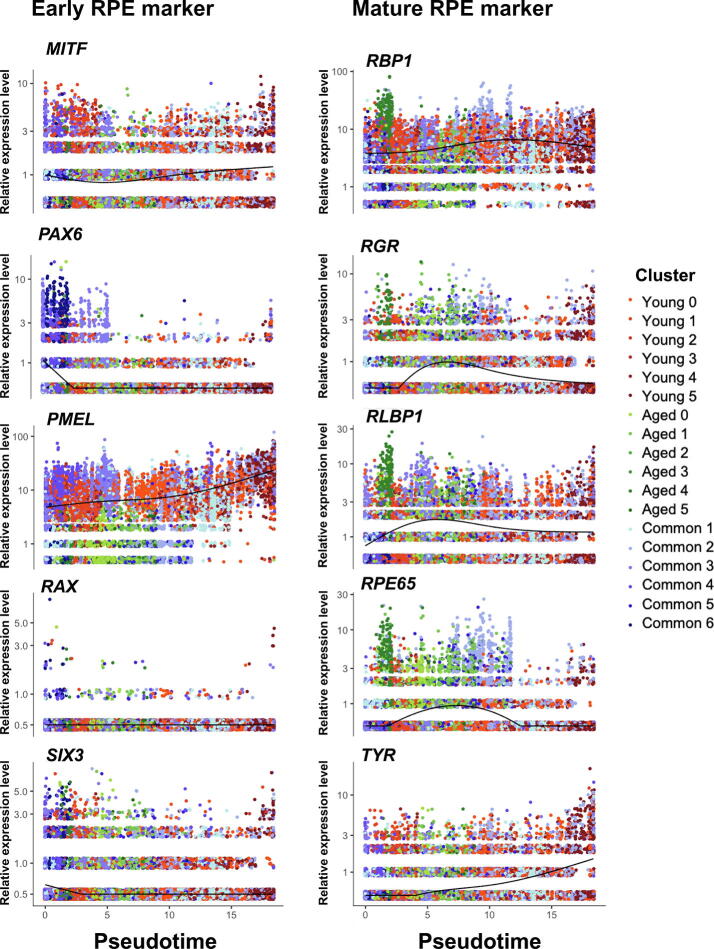
G**ene trajectory by pseudotime analyses of early and mature RPE markers**. Gene expression (measured as normalised UMI count) of early (*MITF*, *PAX6*, *PMEL*, *RAX*, and *SIX3*) and mature RPE markers (*RBP1*, *RGR*, *RLBP1*, *RPE65*, and *TYR*) using Monocle 3, with each subpopulation coded in colour as indicated. Trend of expression is represented by a black line within each panel.

Data dimensionality was reduced with principal component analysis (PCA). Subsequently, the 30 most statistically significant PCs were reduced to two dimensions using the Uniform Manifold Approximation and Projection (UMAP) and used to construct a shared nearest neighbour (SNN) graph for each cell. The Louvain method for community detection was then used to identify clusters in each dataset with resolutions ranging from 0 to 1.5. The results for all resolutions were plotted using clustree [76], which showed the stabilisation of cell population identities at the resolution of 0.6 in the 1-month culture and 0.7 in the 12-month culture (Figure S2A–C). Datasets at both time points were combined into one dataset for comparative analysis with the integration workflow from Seurat [77]. This workflow used canonical correlation analysis (CCA) to identify 22,023 anchors based on 3000 most variable genes. The anchors were then used to align both datasets. To integrate the clusters across both time points, the unsupervised version of MetaNeighbor [17] was used to evaluate the similarities between the 1-month clusters and 12-month clusters. Cluster pairs that were reciprocally top hits and received a mean area under the receiver operating characteristic (AUROC) score greater than 0.8 were merged into one cluster (Figure S2D).

### Cluster characterization and analysis

Network analysis was performed on differentially expressed genes (DEGs) using Reactome functional interaction analysis [78], [79]. Differential expression (DE) analysis was performed using the FindMarkers function based on the likelihood ratio test adapted for single-cell gene expression [80]. GO analysis [81], [82] was performed using a PANTHER overrepresentation test [Fisher exact test, false discovery rate (FDR) < 0.05] against the *Homo sapiens* genome (PANTHER version 14.1 released 2019-03-12). For some clusters, insufficient gene markers are available for GO analysis (Aged Clusters 0, 1, and 5). Canonical RPE markers, gene expression profiles, and their associated GO terms specific to each cluster are provided in Tables S3–S5.

### Trajectory analysis

Trajectory analysis was performed with *Monocle 3* v0.2.4 [52]. Harmonised Pearson residuals produced by the integration step underwent dimensionality reduction with UMAP, and the resulting projection was used to initialize trajectory inference. The node closest to cells expressing proliferative markers was selected as the root of the trajectory, and pseudotime values were calculated. Gene expression dynamics across the trajectory was characterised with Moran I’s test, which was applied via the “*graph_test*” function using the following arguments: neighbor_graph = “principal_graph”, reduction_method = “UMAP”, and expression_family = “quasiposson”. DEGs with FDR ≤ 0.05 were clustered into co-expression modules using the “*find_gene_modules*” function, and the resulting protein interactions were characterised with STRING.

## Ethical statement

The experimental work was approved by the Human Research Ethics Committee of the University of Melbourne (1545484), with the requirements of the National Health and Medical Research Council (NHMRC) of Australia in accordance with the Declaration of Helsinki.

## Code availability

Code and usage notes are available at: https://github.com/powellgenomicslab/RPE_scRNA_AgedStudy. This repository consists of code used to process raw sequencing data in FASTQ format to cell-gene expression tables via the Cell Ranger pipeline, and code used to perform the following analyses: quality control, normalisation, dimensionality reduction, clustering, differential expression, and integration.

## Data availability

Sequencing data are available at ArrayExpress (ArrayExpress: E-MTAB-8511). Files are raw FASTQ files, and a tab separated matrix of UMIs per gene for each cell passing quality control filtering. BAM files can be generated using the supplied repository to process the FASTQ files via Cell Ranger.

## CRediT author statement

**Grace E. Lidgerwood:** Conceptualization, Data curation, Formal analysis, Visualization, Funding acquisition, Investigation, Methodology, Project administration, Resources, Writing - original draft, Writing - review & editing. **Anne Senabouth:** Conceptualization, Data curation, Formal analysis, Visualization, Methodology, Investigation, Software, Writing - original draft, Writing - review & editing. **Casey J.A. Smith-Anttila:** Conceptualization, Methodology, Investigation, Writing - review & editing. **Vikkitharan Gnanasambandapillai:** Conceptualization, Methodology, Investigation, Writing - review & editing. **Dominik C. Kaczorowski:** Conceptualization, Methodology, Investigation, Writing - review & editing. **Daniela Amann-Zalcenstein:** Conceptualization, Methodology, Investigation, Writing - review & editing. **Erica L. Fletcher:** Conceptualization, Methodology, Funding acquisition, Writing - review & editing. **Shalin H. Naik:** Conceptualization, Methodology, Funding acquisition, Writing - review & editing. **Alex W. Hewitt:** Conceptualization, Funding acquisition, Methodology, Resources, Writing - review & editing, Supervision. **Joseph E. Powell:** Conceptualization, Data curation, Formal analysis, Funding acquisition, Methodology, Resources, Software, Writing - original draft, Writing - review & editing, Supervision. **Alice Pébay:** Conceptualization, Formal analysis, Visualization, Funding acquisition, Methodology, Project administration, Resources, Writing - original draft, Writing - review & editing, Supervision. All authors read and approved the final manuscript.

## Competing interests

The authors have declared no competing interests.

## Supplementary material

Supplementary Table S1 **Quality control parameters.**

Supplementary Table S2 **Cluster analysis with number of cells per subpopulation.**

Supplementary Table S3 **Conserved cluster markers, PANTHER Gene Ontology pathways and differential expression for each subpopulation within the “Common” population.**

Supplementary Table S4 **Conserved cluster markers and Gene Ontology pathways for each subpopulation within the “Young” population.**

Supplementary Table S5 **Conserved cluster markers and Gene Ontology pathways for each subpopulation within the “Aged” population.**

Supplementary Table S6 **Monocle3 residual trajectory.**

Supplementary Table S7 **Details of cell filtration criteria.**
